# Overexpression of CD98 in intestinal epithelium dysregulates miRNAs and their targeted proteins along the ileal villus-crypt axis

**DOI:** 10.1038/s41598-018-34474-9

**Published:** 2018-11-01

**Authors:** Moon K. Han, Mark Baker, Yuchen Zhang, Chunhua Yang, Mingzhen Zhang, Pallavi Garg, Emilie Viennois, Didier Merlin

**Affiliations:** 1 Institute for Biomedical Sciences, Center for Diagnostics and Therapeutics, Center for Inflammation, Immunity and Infection, Digestive Disease Research Group, Georgia State University, Atlanta, 30303 USA; 2 Atlanta Veterans Affairs Medical Center, Decatur, 30033 USA

## Abstract

CD98 has been implicated in the experimental model of inflammatory bowel disease. We have previously shown that IEC-specific overexpression of CD98 mediates intestinal inflammation and intestinal epithelial barrier dysfunction. Mice overexpressing CD98 exhibited severe colitis and a greater susceptibility to CAC. Here we demonstrated CD98 overexpression to dysregulate homeostatic gradient profile of miRNA and protein expression along the ileal villus-crypt axis. Using miRNA-target gene prediction module, we observed differentially expressed miRNAs to target proteins of villus and crypt profoundly affected by CD98 overexpression. We have utilized online bioinformatics as methods to further scrutinize the biological meanings of miRNA-target data. We identified significant interactions among the differentially regulated proteins targeted by altered miRNAs in Tg mice. The biological processes affected by the predicted targets of miRNAs deviate from the homeostatic functions of the miRNA-gene-protein axis of the wildtype mice. Our results emphasize a dynamic perturbation of miRNA and protein expression in villus-crypt axis contributing to potential biological consequences of altering CD98 expression. Our findings also suggest the need for a consideration of arrays of interacting biological entities (i.e. miRNAs-mRNAs, protein-protein interaction) or a combination comparison for a better understanding of the disease pathology which is necessary for an effective therapeutic target development.

## Introduction

CD98, a heterodimer of an L-type amino acid transporter (LAT) and β-integrin^1^, has been implicated in the experimental colitis model that mirrors the clinical symptoms of inflammatory bowel disease (IBD). Initially detected in activated immune cells^2,3^, CD98 is constitutively expressed on the epithelial cells of various tissues^4–7^. During chronic intestinal inflammation, a greater number of CD98-positive mononuclear cells^8^ and higher CD98 mRNA levels are found in circulation^9^. Moreover, increased CD98 protein expression^10,11^ is detected in the colonic biopsies of IBD patients. We previously reported that mice with intestinal epithelial cell (IEC)-specific overexpression of CD98 exhibited an exacerbations of colitis and increased susceptibility to colonic tumorigenesis compared to the wild-type mice (WT)^12,13^. This augmented expression of CD98 in the intestinal epithelium (IE) increased intestinal barrier permeability, upregulated pro-inflammatory cytokines, elevated the phosphorylation of proteins associated with β-integrin signaling, and enhanced the expressions of proliferation markers in the small intestine and colonic epithelium^13^. In addition, CD98 overexpression in IE resulted in the aggressive formation of larger and more numerous tumors, suggesting that CD98 may be involved in oncogenesis^14^. In a mouse model that spontaneously develops intestinal carcinomas, greater CD98 expression in IECs enhanced the incidences of small intestinal and colonic tumors, increased cell proliferation, and decreased cell apoptosis^15^.

We speculate the dysregulation of protein expression along the IE upon changes in CD98 expression to be facilitated by microRNAs (miRNAs), which have been implicated in regulating the inflammatory conditions of IBD and various cancers^16–18^. miRNAs are small-noncoding RNAs that target 3′ end of the untranslated regions of their target mRNAs to destabilize gene and protein expression^19,20^. Studies of colonic mucosa of IBD patients have demonstrated miRNA regulation of cellular and humoral immunity^16,21,22^ and cellular processes such as proliferation, apoptosis, extracellular matrix (ECM) organization, cell adhesion, cell surface marker gene expression, oxidative stress, and cellular stress responses^23^. In the colonic biopsies of ulcerative colitis patients, the expression level of miRNA-192 was negatively associated with that of the expression of target genes encoding epithelial-derived chemokines^22^, whereas a dysregulation of miRNAs related to apoptosis has been detected in colonic epithelial cells during experimental colitis^24^. Furthermore, changes in certain miRNAs in non-inflamed mucosal tissue seem to heighten the risk for severe inflammation and cellular changes by associating with genes of cell division, autophagy, apoptosis, ECM organization, cell adhesion, and unfolded protein response^23^.

CD98 modulates the expression levels of certain miRNAs and mRNA products during experimental colitis. An augmentation of IEC-specific CD98 expression levels have an insignificant effect on the basal miRNA expression profile in colon, but differentially modify certain miRNAs under DSS (dextran sulfate sodium) treatment^25^, perhaps synergizing with a preexisting or accompanying aggravator, as such as DSS or inflammation. As IBD and intestinal inflammation are not exclusive to the colon, it is not unconventional to suspect the contribution of altered miRNA and protein expression in various parts of the gastrointestinal tract to the IBD-associated inflammation and pathology. Together with the phenomena of aggravated inflammation in colon and increased incidence of colitis-associated cancer (CAC) under the influence of CD98 overexpression in the experimental setting, a closer look at the effect of CD98 on miRNA-gene-protein axis in IE is needed.

Here, we used publicly available online bioinformatics to comprehensively examine novel data obtained from the mass profiling of gene and protein expression of the ileal villus and crypt under IEC-specific CD98 overexpression.

## Results

### Overexpression of CD98 in IECs alters the expression level of proteins associated with villus and crypt

We previously characterized a mouse model genetically modified to express human CD98 (hCD98) in IECs to study experimental colitis and CAC^13^. Phenotypically, these FVB villin-hCD98 transgenic (Tg) mice were generally healthy; however, they were leaner than FVB WT mice and were found to have smaller microvilli under the electron microscopy^13^. High level of hCD98 (by immunohistochemistry) is strongly expressed in the differentiated cells of the villus and terminally differentiating cells of the crypt of the small intestine^13^. CD98 protein is also abundantly expressed in all sections of the small intestine (duodenum, jejunum, ileum) as indicated by western blot^13^.

**Figure 1:**
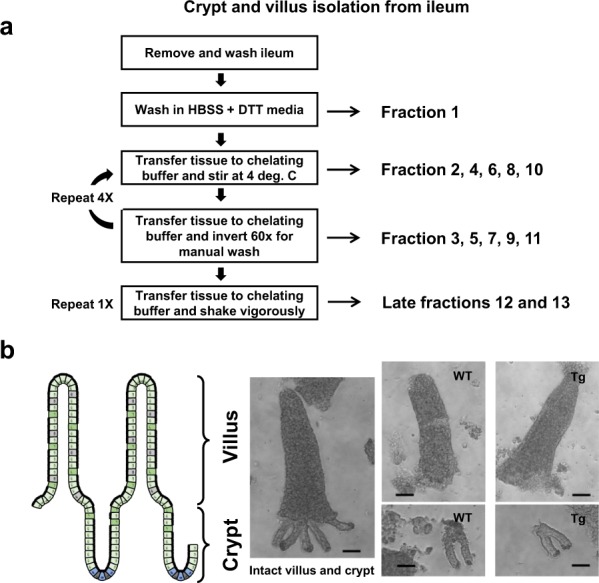
Isolation of ileal epithelial cells from villus and crypt fractions. Ileal epithelial cells were isolated from villi and crypt of FVB (WT) and FVB Villin-hCD98 (Tg) mice at 6–9 weeks of age using a previously described method with modification (**a**). Removed tissue were washed in series of buffer and collected to yield fractions with gradient presence of villi, villi-crypt, or crypt. Fractions were visualized by light microscopy at 10x magnification (**b**, scale bar: 100 µm).

**Figure 2:**
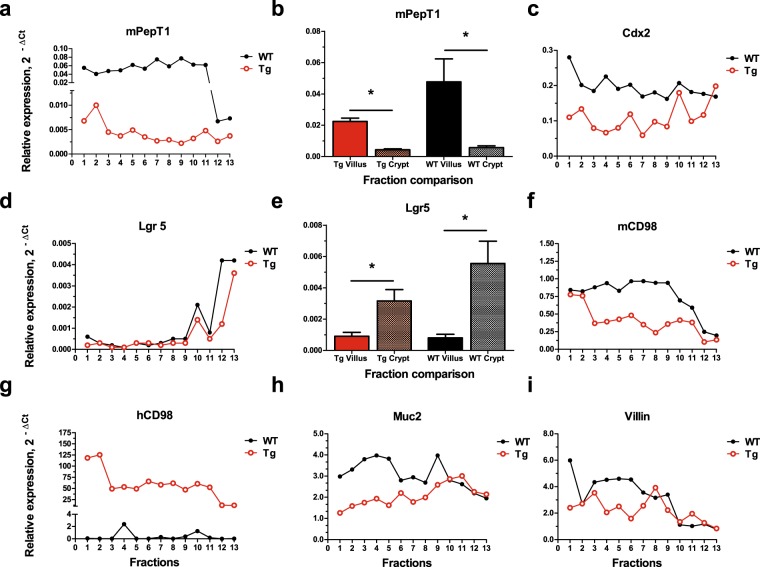
Proteins associated with villus and crypt are disrupted in CD98 overexpressing mice. Total RNA were extracted and pooled from ileal villi and crypt fractions from IEC-specific hCD98 overexpressing transgenic (Tg) and FVB wildtype (WT) mice. Expression levels of villus marker, mPepT1, were examined from different villus and crypt fractions (**b**, n = 6; WT villus vs WT crypt: t = 3.095, P < 0.0102; Tg villus vs Tg crypt: t = 8.346, P < 0.001) with representative expression levels shown across all fractions (**a**). Expression level of crypt marker, Lgr5, was also assessed (**e**, n = 6; WT villus vs WT crypt: t = 3.046, P < 0.0111; Tg villus vs Tg crypt: t = 3.541, P < 0.0046) with representative expression levels shown across all fractions (**d**). Other markers associated with villus and crypt were also examined across all fractions collected (**c**, Cdx2; **f**, mCD98; **g**, hCD98; **h**, Muc2; **i**, Villin).

To evaluate the successful isolation of villus and crypt from the IE, a procedure was modified^26^ (Fig. 1a). The presence of villus and/or crypt in fractions were visualized and confirmed by light microscopy (Fig. 1b). Collected fractions exhibited a gradient of villus, villus-crypt, and crypt. Total RNA was isolated from all fractions (Number of animals: Tg, n = 6; WT, n = 6), and real-time PCR was used to determine the presence of primary villus and crypt markers, mPepT1 and Lgr5, respectively (Fig. 2a,b,d,e). In ileal IE, PepT1 is highly expressed among micro-villus-enriched cells but far more weakly in crypt cells^14,27^. Figure 2b,e show representative findings for the relative mRNA expression levels of mPepT1 and Lgr5, respectively, in all 13 fractions from WT and Tg ileum. The mRNA expression of mPepT1 was greater in WT than Tg across all fractions, with more drastic differences seen in the earlier than the later fractions (Fig. 2a). The mRNA expression pattern of Lgr5 across all fractions was similar in WT and Tg (Fig. 2d). Overall, mPepT1 (villus marker) expression was more evident in the earlier fractions, while Lgr5 (crypt marker) expression was more detectable in the later fractions. When selected fractions were grouped as villus or crypt, we found significant between-region differences for both WT and Tg mice in the analyses of mPepT1 (Fig. 2b, n = 6; WT villus vs. WT crypt: t = 3.095, P < 0.0102; Tg villus vs. Tg crypt: t = 8.346, P < 0.001) and Lgr5 (Fig. 2e, n = 6; WT villus vs. WT crypt: t = 3.046, P < 0.0111; Tg villus vs. Tg crypt: t = 3.541, P < 0.0046). Furthermore, there was no significant statistical difference in the Lgr5 mRNA expression between the crypts of two genotypes (n = 6, t-statistics = 1.492, p-value = 0.150, data not shown). These results indicate that villus and crypt samples were successfully isolated from the ileal IE of WT and Tg. Notably, other markers associated with villus and crypt cells exhibited alterations in Tg (Fig. 2c, Cdx2; 2f, mCD98; 2g, hCD98; 2h, Muc2; 2i, Villin), suggesting disruption to protein expression in villus and crypt due to CD98 overexpression in IE.

### There is a distinctive protein profile along the ileal villus-crypt axis in WT mice

To investigate the protein expression profile along the villus-crypt axis in mouse ileum, we used 2D-DIGE to analyze protein expression in the pooled villus or crypt samples (n = 4 for WT and Tg) within the genotype WT or Tg (i.e. villus vs. crypt) and between WT and Tg (i.e. WT villus vs. Tg villus). For clarification, expression “villus-crypt axis” or “axis” is used when referring to the analysis within the same genotype. As opposed to the conventional expression of “crypt-villus” axis, the term is adopted for this paper due to measurements reflecting the expression in the villus relative to crypt.

Compared spots exhibiting a volume ratio difference of ≥ or ≤2-fold difference in any comparisons were considered differentially expressed, and thus included in the analysis. Gel image analysis was performed using the Differential In-Gel Analysis and Biological Variation Analysis modules of the DeCyder software. The following results were obtained for the number of differentially expressed gel spots for each comparison (Supplementary Table S1): 115 upregulated and 106 downregulated spots in WT villus-crypt axis; 140 upregulated and 226 downregulated spots in Tg villus-crypt axis; 30 upregulated and 63 downregulated spots in Tg villus compared to WT villus; 39 upregulated and 30 downregulated spots in Tg crypt compared to WT crypt (cropped gel images in Supplementary Fig. S1.1; Originals in Supplementary Fig. S1.2).

Due to the sheer abundance of a number of differentially expressed proteins and the limitation of spot selection such as the wide range of fold differences qualified for analysis consideration and/or the location in 2D gel, we sought to qualitatively decrease the pool of spots that would be considered for further analysis. Exclusion/inclusion criteria were based first on identifying the spots that had undergone a potential protein modification and/or spots that were present or absent in gel comparisons. Of a total of 719 spots that were differentially expressed across various comparisons, we selected 318 spots using the above criteria. From them, 20 spots common to both axial (within-genotype) comparisons, 8 spots from the villus comparison, and 7 spots from the crypt comparisons were chosen based on their fold changes and location in the gel (Supplementary Table S1, Figs S2 and S3). Some spots were common to all comparisons. In total, 32 independent spots were picked from all four comparisons for further evaluation.

**Figure 3:**
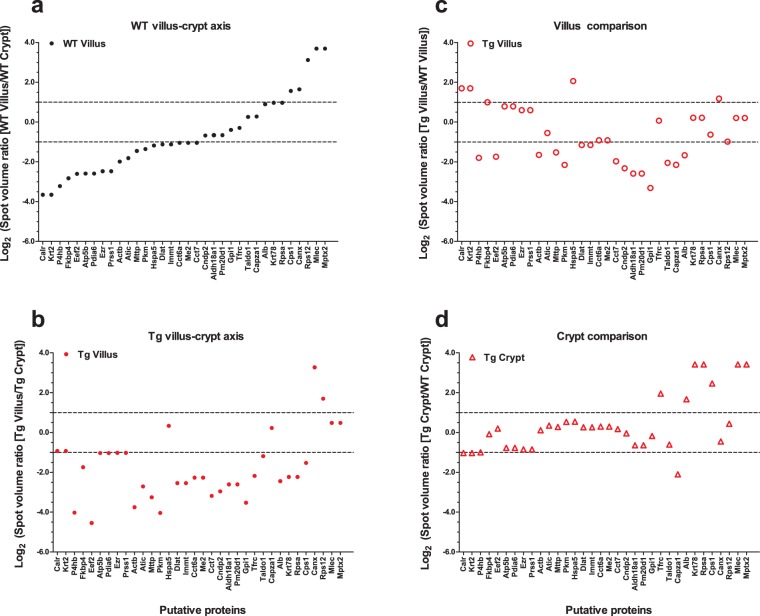
Distribution of differentially expressed proteins is altered in CD98 overexpressing mice. Putative proteins from 32 spots were identified using LC-MS and were considered differentially expressed if the level fold change is ≥2. Collectively from all four comparisons, 34 different putative proteins (labeled in figure as genes responsible for encoding them) were identified where, among the repeats, only the putative protein with the highest score is included. Putative proteins in the villus of WT (**a**) and Tg (**b**) were compared to its respective crypt to determine their distribution profile along the axis. Cross-comparison between the genotypes were also made between the villi of WT and Tg (**c**) and between the crypt of WT and Tg (**d**). Dotted lines indicate the threshold for 2 fold change. Upper = (+) 2-fold change; Lower = (−) 2-fold change. Plots between the threshold lines indicate proteins with less than 2 fold change, thus a minimal expression difference, for the comparison. Y-axis is in Log_2_ (Spot volume ratio) scale.

To identify the putative proteins corresponding to the 32 selected spots, we performed LC-MS. The search returned 27 putative uncharacterized proteins and 133 accession IDs that corresponded to 101 different proteins. We selected the top two putative proteins identified for each spot (unless only one was identified), as ranked by protein score and the number of unique peptides. This analysis yielded 62 proteins, several of which were identified multiple times (Table 1 and Supplementary Table S2.1 and S2.2). From the four comparisons, we identified 34 different putative proteins (Table 1 and Fig. 3, labeled as their encoding genes). When a protein was repeatedly identified, we included only the candidate with the highest score in relevant tables and figures. To determine the distribution of the selected putative proteins along the axis, we examined their levels in villus relative to its crypt. Of the 34 proteins, 19 were downregulated and 5 were upregulated in the villus compared to crypt in the WT (Table 1 and Fig. 3a), and 10 showed only minimal changes of less than 2-fold difference (plots located inside the 2-fold threshold dotted lines, Fig. 3a). Our results indicate that WT ileal epithelium exhibits a certain distribution gradient of specific proteins along the villus-crypt axis.

**Table 1:** Distinctive protein profile along the ileal villus-crypt axis is disrupted in CD98 overexpressing mice.

Spot No.^a^	Accession ID^b^	Protein Name	Gene	Score^c^	No. of Unique Peptides^d^	MW [kDa]	Protein pI^e^	Relative expression Log_2_ (Spot Volume Ratio)			
								WT Villus-Crypt Axis	Tg Villus-Crypt Axis	Between Villus (Tg: WT)	Between Crypt (Tg: WT)
719	Q3UWP8	Calreticulin (calregulin)	Calr	10.40	2	42.20	4.74	−3.66	−0.93	1.69	−1.03
719	B2RTP7	Krt2 protein	Krt2	10.97	2	70.90	8.06	−3.66	−0.93	1.69	−1.03
619	Q3UDR2	Prolyl 4-hydroxylase, beta polypeptide	P4hb	122.95	10	56.60	4.89	−3.22	−4.02	−1.80	−1.00
550	P30416	Peptidyl-prolyl cis-trans isomerase FKBP4	Fkbp4	41.62	5	51.50	5.72	−2.83	−1.75	0.99	−0.09
150	P58252	Elongation factor 2	Eef2	294.38	26	95.30	6.83	−2.60	−4.55	−1.74	0.20
726	P56480	ATP synthase subunit beta, mitochondrial	Atp5b	96.05	7	56.30	5.34	−2.59	−1.04	0.79	−0.77
726	Q922R8	Protein disulfide-isomerase A6	Pdia6	53.55	6	48.10	5.14	−2.59	−1.04	0.79	−0.77
263	Q8CBU4	Ezrin (cytovillin)	Ezr	15.91	2	50.20	5.80	−2.47	−1.03	0.59	−0.85
263	Q9Z1R9	MCG124046 (Protease, Serine, 1)	Prss1	26.44	1	26.10	4.94	−2.47	−1.03	0.59	−0.85
143	Q6IWE2	Beta-actin (Fragment)	Actb	14.51	1	8.60	4.49	−1.99	−3.75	−1.65	0.11
424	Q9CWJ9	Bifunctional purine biosynthesis protein PURH	Atic	104.14	10	64.20	6.76	−1.82	−2.71	−0.55	0.35
144	O08601	Microsomal triglyceride transfer protein large subunit	Mttp	45.99	8	99.00	7.62	−1.46	−3.26	−1.52	0.28
526	P52480	Pyruvate kinase PKM	Pkm	65.83	7	57.80	7.47	−1.36	−4.04	−2.15	0.53
313	P20029	Heat shock protein 5	Hspa5	104.65	9	72.40	5.16	−1.19	0.33	2.06	0.54
399	Q8BMF4	Dihydrolipoyllysine-residue acetyltransferase component of pyruvate dehydrogenase complex, mitochondrial	Dlat	120.64	9	67.90	8.57	−1.13	−2.55	−1.16	0.26
399	E9Q800	Mitochondrial inner membrane protein	Immt	13.63	3	75.60	7.80	−1.13	−2.55	−1.16	0.26
436	P80317	T-complex protein 1 subunit zeta	Cct6a	44.40	4	58.00	7.08	−1.05	−2.27	−0.92	0.30
436	Q99KE1	Malic enzyme 2, mitochondrial NAD-dependent	Me2	39.24	6	65.80	7.61	−1.05	−2.27	−0.92	0.30
527	A0A0N4SV00	T-complex protein 1 subunit eta	Cct7	138.27	11	55.00	7.83	−1.04	−3.18	−1.98	0.17
581	Q9D1A2	Cytosolic non-specific dipeptidase	Cndp2	85.77	10	52.70	5.66	−0.68	−2.96	−2.32	−0.04
556	Q9Z110-2	Isoform Short of Delta-1-pyrroline-5-carboxylate synthase	Aldh18a1	28.93	4	87.00	7.55	−0.66	−2.61	−2.59	−0.64
556	Q8C165	Probable carboxypeptidase PM20D1	Pm20d1	63.73	7	55.60	6.43	−0.66	−2.61	−2.59	−0.64
88	B2RXT5	Glucose-6-phosphate isomerase	Gpi1	1.56	1	38.30	8.48	−0.40	−3.53	−3.32	−0.18
178	Q8C872	Transferrin receptor protein 1	Tfrc	5.22	1	57.30	6.48	−0.30	−2.18	0.07	1.95
1031	Q93092	Transaldolase 1	Taldo1	34.64	4	37.40	7.03	0.25	−1.19	−2.05	−0.61
1025	P47753	F-actin-capping protein subunit alpha-1	Capza1	39.84	4	32.90	5.55	0.27	0.22	−2.15	−2.10
176	P07724	Serum albumin	Alb	17.61	2	68.60	6.07	0.89	−2.45	−1.67	1.67
1336	A1L0X5	Krt78 protein (Fragment)	Krt78	17.62	1	54.70	6.30	0.96	−2.24	0.21	3.41
1336	B2CY77	Laminin receptor (Fragment)	Rpsa	45.76	4	32.80	4.87	0.96	−2.24	0.21	3.41
167	Q561M8	Cps1 protein (Fragment)	Cps1	6.81	1	32.20	9.25	1.56	−1.53	−0.63	2.46
501	P35564	Calnexin	Canx	29.94	3	67.20	4.64	1.64	3.27	1.17	−0.45
1847	P63323	40S ribosomal protein S12	Rps12	12.78	1	14.50	7.24	3.12	1.69	−0.99	0.43
1406	Q6ZQI3	Malectin	Mlec	17.11	2	32.30	6.05	3.69	0.48	0.20	3.42
1406	D3YYJ7	Pentaxin	Mptx2	5.58	1	24.50	8.90	3.69	0.48	0.20	3.42

Of the repeated protein candidates, only the putative protein with highest score is shown in this table. Repeats are shown in Supplementary Table S2.1 and S2.2. Average fold change in axis refers to changes in villus compared to its respective crypt within same genotype. Average fold change between the villi or the crypt refers to changes between two different genotypes. (−) Sign refers to downward fold change.

^a^Spot Number: Spot identity in gel.

^b^Accession ID: Unique identifier of the protein based on FASTA database.

^c^Score: Total score of the protein based on individual peptides.

^d^Unique peptides: Number of peptide sequence unique to the protein group.

^e^Protein pI: Theoretical isoelectric point based on protein molecules.

### Overexpression of CD98 in IECs disturbs protein expression along the villus-crypt axis of the ileal epithelium

To investigate the effect of IEC-specific CD98 overexpression along the IE on protein expression, we scrutinized the same 34 putative proteins along the axis of Tg mice. Of them, 26 were downregulated and 2 were upregulated in the villus compared to its crypt in Tg, (Table 1 and Fig. 3b), while 6 showed only minimal changes (Fig. 3b). An overlay of differentially expressed proteins in the axes of WT and Tg showed, that villus expression level of 9 proteins were above (red circles to the left of black triangle plots) and 24 proteins were below (red circles to the right of black plots) in the Tg villus-crypt axis compared to that of WT axis (Supplementary Fig. S4). Targets *Capza1* and *P4hb* showed minimal CD98 overexpression-related changes along the axis (Supplementary Fig. S4).

The relative expression levels in villus compared to its crypt has revealed disruption to the protein distribution between WT and Tg. While Cndp2, Aldh18a1, Pm20d1, Gpi1, Tfrc, Taldo1, Alb, Krt78, and Rpsa remain minimally varied in its gradient expression along the WT axis, there is a differential expression in Tg axis. On the other hand, differential expression of Calr, Krt2, Hspa5, Mlec, and Mptx2 is shown to be leveled in Tg axis compared to WT axis. These results demonstrate that IEC-specific CD98 overexpression disrupts the homeostatic expression gradients of numerous proteins found along the ileal villus-crypt axis.

### Overexpression of CD98 in IECs changes protein expression levels in villus and crypt cells

To investigate the effect of CD98 overexpression on protein expression in villus and crypt, we compared the levels of 34 identified putative proteins in these regions of WT versus Tg mice. We found that four candidates were upregulated and 15 were downregulated in Tg villus compared to WT villus (red dots outside the 2-fold change threshold line, Fig. 3c). In crypt, overexpression of CD98 upregulated seven putative proteins and downregulated four such proteins in Tg versus WT mice (Fig. 3d). The expression levels of 15 and 23 putative proteins in villus and crypt, respectively, were minimally affected by the genotypic difference (Fig. 3c,d). These results reveal that CD98 overexpression has specific effects on protein levels in the cells of villus and crypt contributing to the differential distribution along the villus-crypt axis we have identified so far.

### The expression profile of intestinal miRNAs differs between ileal IECs of the villus and crypt

To determine if overexpression of CD98 dysregulates the expression patterns of miRNAs in IECs along the axis, we examined a panel of miRNAs from the total RNAs extracted from selected villus and crypt fractions of WT and Tg mice. On average, we detected 243 miRNAs per sample. Across all comparisons (between villus and crypt within the same genotype or between WT and Tg), 214 miRNAs were differentially expressed (cutoff p-value of 0.05). A principal component analysis of the top 50 miRNAs with the largest variation for each group showed a distinct separation of the villus and crypt within WT and Tg, but partial overlaps in clusters when the data by region were plotted (Supplementary Fig. S5a). Two-way hierarchical clustering of the miRNAs further confirmed distinct cluster formations for villus or crypt within a given genotype (Supplementary Fig. S5b).

### Overexpression of CD98 in IECs dysregulates the miRNA expression profile along the villus-crypt axis

**Figure 4:**
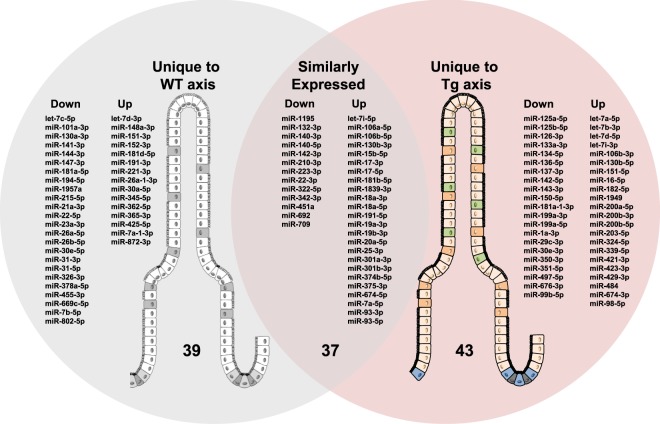
CD98 overexpression in IECs alter unique miRNAs associated with villus-crypt axis profile. All miRNAs listed were found to be differentially expressed using a cutoff p-value of <0.05. A total of 119 miRNAs were differentially expressed along the villus-crypt axis of WT and Tg ileal IECs. Among them, there was an overlap of 37 miRNAs found to be similarly expressed in both genotypes while 39 miRNAs and 43 miRNAs were uniquely distributed along the axis to either WT or Tg, respectively. miRNAs listed reflect the directional expression found in villus compared to crypt along the axis of the same genotype. The left column of each grouping is the downregulated miRNAs, whereas the right column represents upregulated miRNAs in the villus.

**Figure 5:**
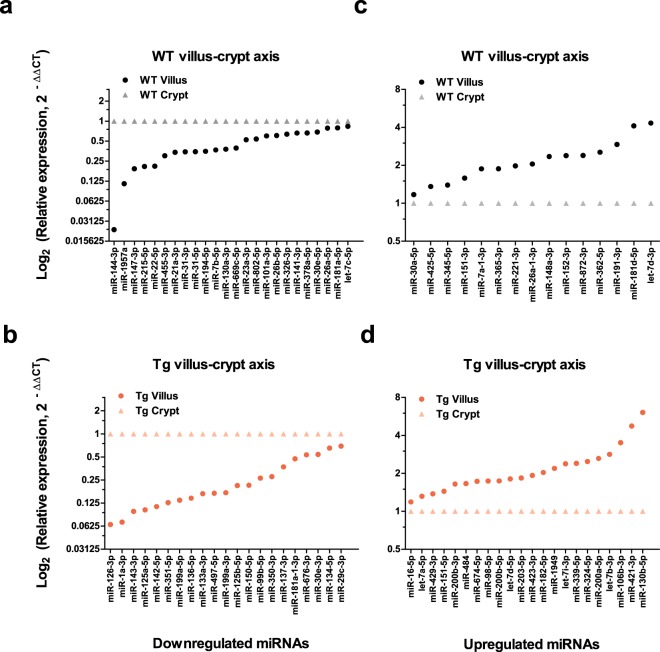
Expression levels of certain miRNAs are uniquely affected by the overexpression of CD98 in IECs. Relative fold change (2^−∆∆CT^) of miRNAs differentially affected by CD98 overexpression is shown. All miRNAs listed were found to be differentially expressed using a cutoff p-value of < 0.05. These miRNAs expression are unique to the axis of either genotype. In WT, 24 miRNAs were downregulated (**a**) and 15 miRNAs were upregulated (**c**) in the villus compared to its crypt. In Tg villus-crypt axis, 21 miRNAs were downregulated (**b**) and 22 miRNAs were upregulated (**d**) in the villus compared to its crypt. Y-axis is shown in Log_2_ (Relative expression) scale.

To determine if miRNA expression profile along the axis differs between the two genotypes, we first examined the differences in normalized miRNA expression (expressed in figures as Log_2_ (fold change, 2^−∆∆CT^)) in villus versus crypt for each genotype, resulting in a villus-crypt axial miRNA expression profile for that genotype. We then compared these profiles between the WT and Tg. Comparison revealed uniquely distributed 119 miRNAs along the villus-crypt axis (Fig. 4). In the ileal epithelium, 76 miRNAs in WT and 80 miRNAs in Tg were differentially expressed in the villus relative to its crypt (Fig. 4). Among them, 37 miRNAs were similarly distributed along the axis in both Tg and WT, while 39 and 43 miRNAs were unique to the WT and Tg, respectively. Of the 37 miRNAs that were similarly expressed in the axis of WT and Tg, 13 were downregulated and 24 were upregulated (Fig. 4 and Supplementary Fig. S6) in the villus relative to crypt. We also identified miRNAs that were uniquely regulated in the axes of WT and Tg (Fig. 5). Of the 39 miRNAs uniquely regulated in WT villus, 24 were downregulated and 15 were upregulated (Figs 4 and 5a,c). Of the 43 miRNAs uniquely regulated in the villus versus crypt of Tg mice, 21 were downregulated and 22 were upregulated (Figs 4 and 5b,d). These results suggest that CD98 overexpression in ileal IECs can alter the distributions of some miRNAs while leaving others relatively unchanged.

### Overexpression of CD98 in IECs alters miRNA expression levels in both villus and crypt cells

**Figure 6:**
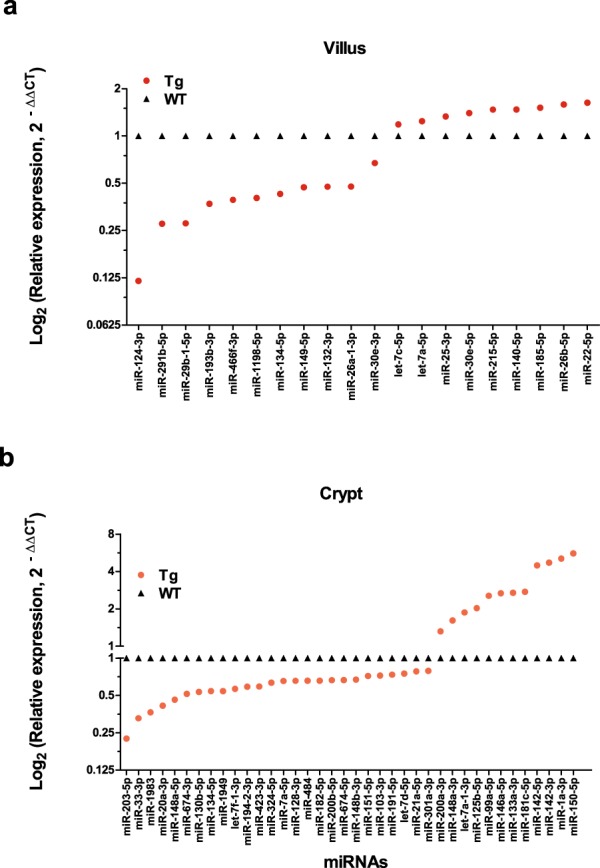
Villus and crypt harbor unique miRNA expression profile which is altered by CD98 overexpression. Relative fold change (2^−∆∆CT^) of miRNAs detected in either villus or crypt of WT and CD98 Tg ileal IECs is shown. To determine if there was a regional variation in miRNA expression profile due to genotypic difference, Tg villi were compared with WT villi (**a**) and Tg crypt were compared with WT crypt (**b**). Of the 58 miRNAs identified from these comparisons, with the exclusion of miR-134-5p, -19 and -37, miRNA profile signature is unique to the villus or crypt. Y-axis is shown in Log_2_ (Relative expression) scale.

To determine if CD98 overexpression altered the miRNA expression profiles in cells of villus and/or crypt, we compared the relative miRNA expression levels of these tissues between WT and Tg animals (Fig. 6, Supplementary Table S3). Twenty miRNAs were differentially expressed in Tg villus relative to WT; of them, 11 were downregulated and 9 were upregulated (Fig. 6a). In crypt, 38 miRNAs were differentially expressed; of them, 26 were downregulated and 12 were upregulated in Tg relative to WT (Fig. 6b). Of the 58 miRNAs identified from these comparisons, 19 and 37 were unique to the villus and crypt comparisons, respectively (the lone shared miRNA was miR-134-5p). These results suggest that CD98-overexpressing mice have different miRNA expression profiles in their villus and crypt regions, relative to WT mice, contributing to the genotype-related differences observed in miRNA distributions along the axis.

### Putative proteins that are differentially expressed in villus and crypt of Tg mice are targeted by the miRNAs that are uniquely affected by CD98 overexpression

Next, the target genes of 119 miRNAs found to be differentially expressed along the axis were predicted using the Predicted Target Module (microRNA-gene targets) of miRWalk 2.0^28^. Then we examined for any overlaps between the potential targets with the differentially expressed 34 putative proteins selected above. Of the 119 miRNAs, seven *let-7*-related miRNAs were excluded from the target assessment. According to our analysis, 63 miRNAs were predicted to target 29 of the 34 putative proteins identified in our proteomic analysis. The 5 that did not yield any matches were those encoded by *Cct7*, *Aldh18a1*, *Alb*, *Rps1*2, and *Mptx*. Among the 63 miRNAs, 32 had a single genomic target, while 31 had multiple targets from the list of 29 proteins in the table (Table 2). Seventeen of the 24 miRNAs that were uniquely downregulated in WT villus versus crypt targeted 20 proteins; Ten of 15 upregulated miRNAs targeted 13 proteins in these mice (Table 3, left). In Tg, 12 of the 21 miRNAs that were uniquely downregulated in villus versus crypt targeted 12 proteins, while 7 of 22 upregulated miRNAs targeted 12 proteins (Table 3, right). Among the miRNAs that were similarly distributed in both axes, 4 of 13 miRNAs downregulated in the villus targeted 6 proteins, and 13 of 24 upregulated miRNAs targeted 11 proteins (Table 3, center).

**Table 2:** Differentially regulated miRNAs in the villus-crypt axis have genomic targets among the putative proteins in which the expression profile was affected by CD98 overexpression.

Putative targets	miRNAs targeting the putative target gene		# of miRNAs
	Single Target	Multiple Targets	
Prss1		miR-17-3p	1
Gpi1		miR-425-5p, miR-150-5p	2
Eef2	miR-322-5p, miR-497-5p	miR-709, miR-326-3p, miR-484	5
Actb	miR-132-3p, miR-1a-3p	miR-18a-5p, miR-802-5p	4
Mttp		miR-30a-5p, miR-30e-5p	2
Cps1	miR-151-5p		1
Krt78	miR-31-5p, miR-134-5p	miR-199a-5p	3
Tfrc	miR-7a-5p, miR-221-3p, miR-365-3p, miR-7b-5p	miR-30a-5p, miR-30e-5p, miR-130b-5p	7
Ezr		miR-17-3p	1
Hspa5	miR-374b-5p, miR-350-3p	miR-130b-3p, miR-301a-3p, miR-351-5p, miR-130a-3p, miR-148a-3p, miR-152-3p, miR-125a-5p, miR-125b-5p, miR-150-5p, miR-351-5p	12
Immt	miR-191-3p	miR-342-3p, miR-802-5p, miR-203-5p	4
Dlat	miR-191-5p, miR-194-5p, miR-872-3p	miR-421-3p	4
Atic	miR-30e-3p, miR-455-3p	miR-18a-5p, miR-342-3p, miR-709, miR-326-3p	6
Me2	miR-106a-5p, miR-17-5p, miR-93-5p	miR-351-5p, miR-101a-3p, miR-144-3p, miR-125a-5p, miR-125b-5p, miR-203-5p	9
Cct6a	miR-26a-5p, miR-26b-5p	miR-101a-3p, miR-144-3p, miR-7a-1-3p, miR-130b-5p	6
Canx		miR-130b-3p, miR-301a-3p, miR-130a-3p, miR-144-3p, miR-148a-3p	5
P4hb		miR-199a-5p, miR-484	2
Pkm	miR-378a-5p	miR-130b-5p	2
Atp5b		miR-23a-3p	1
Fkbp4	miR-21a-3p, miR-137-3p	miR-181a-5p, miR-181d-5p	4
Pm20d1		miR-143-3p, miR-674-3p	2
Cndp2		miR-351-5p, miR-709, miR-125a-5p, miR-125b-5p, miR-351-5p, miR-674-3p	6
Krt2		miR-23a-3p	1
Calr	miR-22-5p	miR-709, miR-148a-3p, miR-152-3p, miR-143-3p	5
Pdia6	miR-106b-5p, miR-20a-5p	miR-181a-5p, miR-181d-5p, miR-23a-3p	5
Capza1		miR-181a-5p, miR-181d-5p, miR-23a-3p, miR-30a-5p, miR-30e-5p, miR-7a-1-3p	6
Taldo1	miR-19b-3p	miR-130b-3p, miR-301a-3p, miR-130a-3p	4
Rpsa		miR-425-5p	1
Mlec	miR-399-5p	miR-421-3p, miR-674-3p	3
**Total**	**29**	**32**	**31**

miRNAs listed in each row represents miRNAs targeting the putative target gene listed in the corresponding row. miRNAs listed under “Single Target” refer to miRNAs that solely target the gene in the same row, whereas the miRNAs listed under “Multiple Targets” refer to miRNAs targeting genes listed elsewhere in the table, including the one it is listed under. Values in very far right panel refer to the number of miRNAs targeting the putative gene in the corresponding row.

**Table 3:** Potential genomic targets of differentially and similarly expressed miRNAs in ileal villus-crypt axis of WT and Tg.

Unique to WT axis				Similarly expressed in WT and Tg				Unique to Tg axis			
Genomic targets^a^	miRNAs	Genomic targets^b^	miRNAs	Genomic targets^a^	miRNAs	Genomic targets^b^	miRNAs	Genomic targets^a^	miRNAs	Genomic targets^b^	miRNAs
Actb	miR-802-5p	Calr	miR-152-3p	Actb	miR-132-3p	Actb	miR-18a-5p	Actb	miR-1a-3p	Cct6a	miR-130b-5p
Atic	miR-455-3p		miR-148a-3p	Atic	miR-709	Atic	miR-18a-5p	Atic	miR-30e-3p	Cndp2	miR-674-3p
	miR-326-3p	Canx	miR-148a-3p		miR-342-3p	Canx	miR-301a-3p	Calr	miR-143-3p	Cps1	miR-151-5p
Atp5b	miR-23a-3p	Capza1	miR-7a-1-3p	Calr	miR-709		miR-130b-3p	Cndp2	miR-351-5p	Dlat	miR-421-3p
Calr	miR-22-5p		miR-30a-5p	Cndp2	miR-709	Dlat	miR-191-5p		miR-125b-5p	Eef2	miR-484
Canx	miR-144-3p		miR-181d-5p	Eef2	miR-709	Ezr	miR-17-3p		miR-125a-5p	Immt	miR-203-5p
	miR-130a-3p	Cct6a	miR-7a-1-3p		miR-322-5p	Hspa5	miR-374b-5p	Eef2	miR-497-5p	Me2	miR-203-5p
Capza1	miR-30e-5p	Dlat	miR-872-3p	Immt	miR-342-3p		miR-301a-3p	Fkbp4	miR-137-3p	Mlec	miR-674-3p
	miR-23a-3p	Fkbp4	miR-181d-5p	**6** ^**c**^	**4** ^**d**^		miR-130b-3p	Gpi1	miR-150-5p		miR-421-3p
	miR-181a-5p	Gpi1	miR-425-5p			Me2	miR-93-5p	Hspa5	miR-351-5p		miR-339-5p
Cct6a	miR-26b-5p	Hspa5	miR-152-3p				miR-17-5p		miR-350-3p	P4hb	miR-484
	miR-26a-5p		miR-148a-3p				miR-106a-5p		miR-150-5p	Pkm	miR-130b-5p
	miR-144-3p	Immt	miR-191-3p			Pdia6	miR-20a-5p		miR-125b-5p	Pm20d1	miR-674-3p
	miR-101a-3p	Mttp	miR-30a-5p				miR-106b-5p		miR-125a-5p	Tfrc	miR-130b-5p
Dlat	miR-194-5p	Pdia6	miR-181d-5p			Prss1	miR-17-3p	Krt78	miR-199a-5p	**12** ^**c**^	**7** ^**d**^
Eef2	miR-326-3p	Rpsa	miR-425-5p			Taldo1	miR-301a-3p		miR-134-5p		
Fkbp4	miR-21a-3p	Tfrc	miR-365-3p				miR-19b-3p	Me2	miR-351-5p		
	miR-181a-5p		miR-30a-5p				miR-130b-3p		miR-125b-5p		
Hspa5	miR-130a-3p		miR-221-3p			Tfrc	miR-7a-5p		miR-125a-5p		
Immt	miR-802-5p	**13** ^**c**^	**10** ^**d**^			**11** ^**c**^	**13** ^**d**^	P4hb	miR-199a-5p		
Krt2	miR-23a-3p							Pm20d1	miR-143-3p		
Krt78	miR-31-5p							**12** ^**c**^	**12** ^**d**^		
Me2	miR-144-3p										
	miR-101a-3p										
Mttp	miR-30e-5p										
Pdia6	miR-23a-3p										
	miR-181a-5p										
Pkm	miR-378a-5p										
Taldo1	miR-130a-3p										
Tfrc	miR-7b-5p										
	miR-30e-5p										
**20** ^**c**^	**17** ^**d**^										

miRNAs listed reflect the directional expression found in villus compared to crypt along the axis of WT and Tg, either differentially (WT, left; Tg, right) or similarly expressed (center).

^a^Genomic targets of downregulated miRNAs.

^b^Genomic targets of upregulated miRNAs.

^c^Total number of genomic targets.

^d^Number of different miRNAs.

**Figure 7:**
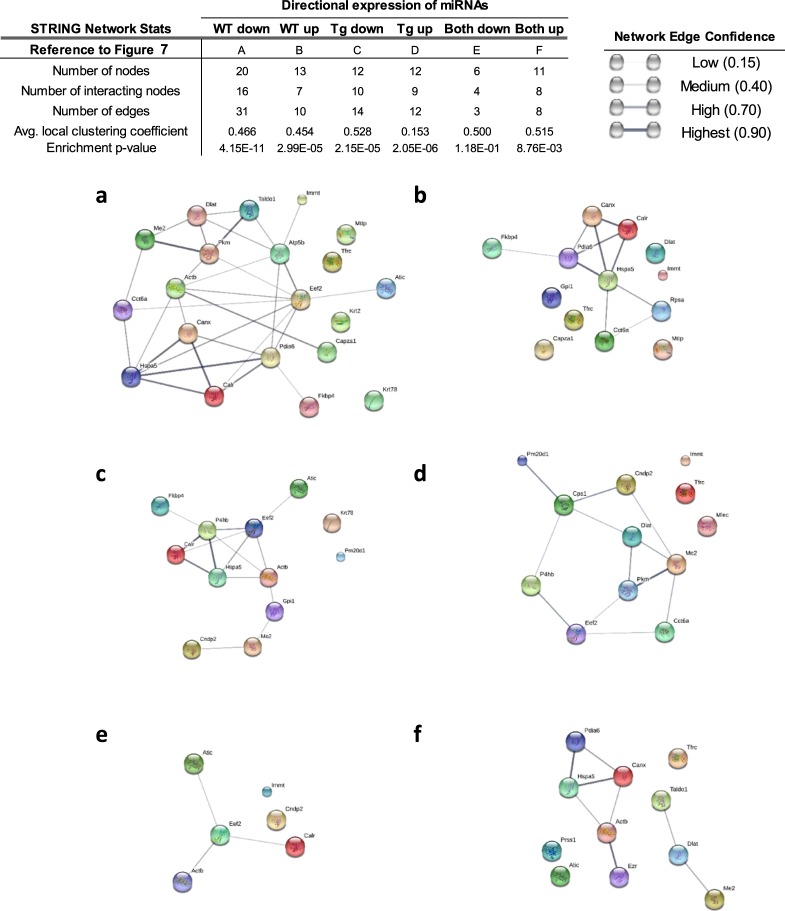
Overexpression of CD98 in IECs dysregulates the interaction of proteins expressed in villus and crypt. Visual representation of STRING network for putative proteins targeted by miRNAs differentially regulated in the ileal villus-crypt axis of WT and Tg is shown. Representation of interactions via network edge lines are shown among proteins/genes targeted by uniquely downregulated or upregulated miRNAs in WT villus (**a**,**b**, respectively) or Tg villus (**c**,**d**, respectively). Interaction is also shown among the proteins/genes targeted by similarly expressed miRNAs in two genotypes (**e**,**f**). Spheres of different colors represent nodes (proteins). While large nodes are indicative of a presence of a known of predicted 3D structure, protein structures for the small nodes are currently unknown. The lines between nodes represent “edges” or protein-protein associations. The thickness and darkness of those edges represent confidence score that is indicative of likeliness of a true interaction based on available evidence. Evidence is ranked 0 to 1 with 1 being the highest likeliness of protein-protein interaction. The location of nodes or the length of edges between the nodes does not indicate the strength of association with the connecting or neighboring nodes.

Having identified potential target proteins in the axes of WT and Tg, we next examined for the presence of protein-protein interaction. To predict and identify functional or physical networks among the proteins, we utilized STRING^29^. The goal was to identify the presence of interactions and the strength of their association in a network based on the evidence curated from various databases employed by the tool. From this analysis, we extracted the information such as the number of interactions (edges) among the proteins targeted by the differentially expressed miRNAs, the strength of each association (network edge confidence), an average measure of the tendency of each protein to connect with other proteins in the network (average clustering coefficient, ACC; 1 = highest, 0 = lowest), and the contribution of proteins to a known biological processes (enrichment p-value) (Fig. 7). Out of 20 putative targets of 17 miRNAs downregulated in WT villus-crypt axis, 16 proteins (connected nodes) were determined to interact with each other (Fig. 7a). This network had a total of 31 interactions among the individual proteins, an ACC of 0.466, and an enrichment p-value of 4.11E-11. Of the 13 predicted targets of 10 miRNAs upregulated in WT axis, 7 proteins showed 10 different interactions to form a network with an ACC of 0.454 and an enrichment p-value of 2.99E-5 (Fig. 7b). Of the 12 proteins targeted by 12 miRNAs downregulated in Tg axis, 14 interactions were seen among 10 proteins (Fig. 7c). The network had an ACC of 0.528 and an enrichment p-value of 2.15E-5. Of 12 proteins targeted by 7 miRNAs upregulated in Tg axis, 9 were predicted to interact (Fig. 7d). This network had a relatively low ACC of 0.153, but a significant enrichment p-value of 2.05E-6. Among the miRNAs that were similarly upregulated in the villus versus crypt in both WT and Tg, 4 miRNAs targeted 6 proteins, 4 of which interacted (Fig. 7e). These 4 proteins had only 3 interactions among them, with a modest ACC of 0.500 and an insignificant enrichment p-value of 1.18E-1. Of the miRNAs that were downregulated to similar degrees in the villus versus crypt in both WT and Tg, 13 miRNAs targeted 11 proteins, 8 of which showed interactions that were separated into 2 clusters (Fig. 7f). This network had a modest ACC of 0.515 and a significant enrichment p-value of 8.76E-3. Together, our findings regarding the predicted interactions among proteins targeted by miRNAs that were differentially expressed in the axes of WT or Tg mice suggest that these targeted downstream products have substantial association. These collective findings may help explain the biological consequences of widespread protein interactions due to CD98 overexpression in IECs.

### Overexpression of CD98 in IECs modulates biological processes by dysregulating multiple miRNA-gene interactions

To investigate biological processes affected by the above-described interactions, we used DAVID^30,31^ to perform functional annotation clustering, which was stratified according to the genotype and the directional changes of miRNA expression in the villus of the axis in question.

The analysis of the interacting proteins targeted by miRNAs that were altered in WT villus versus crypt yielded 4 and 2 clusters for the downregulated and upregulated miRNAs, respectively (Supplementary Table S3). Determined putative targets of the miRNAs downregulated in the WT axis were strongly associated with cadherin binding, cell-cell adhesions and junctions, isopeptide bonds, mitochondria, and endoplasmic reticulum (ER) activities, among others. Associations with cytoplasm, extracellular exosomes, poly(A)-RNA binding and acetylation, ER activities, and melanosomes were found for the putative targets of miRNAs upregulated in the WT axis (Supplementary Table S3).

In Tg villus, the analysis yielded 5 and 3 clusters of interacting proteins targeted by miRNAs that were downregulated and upregulated, respectively (Supplementary Table S4). Functions enriched by the proteins most affected by the downregulated miRNAs were strongly associated with protein folding, chaperones, poly(A)-RNA binding, the cytosol, and Ubl conjugation, among others. Three clusters enriched by the interacting proteins targeted by the upregulated miRNAs were considerably associated with metabolic processes, mitochondria, and proton acceptor and metal ion binding (Supplementary Table S4).

Biological processes enriched by the miRNAs of similar expression levels in villi of both WT and Tg formed 1 and 2 functional clusters of weak association targeted by up- and downregulated miRNAs, respectively (Supplementary Table S5). These results indicate that IEC-specific CD98 overexpression perturbs various biological processes by potentially altering homeostatic miRNA-gene interaction which consequently leads to changes in protein-to-protein interaction.

## Discussion

Our study demonstrates IE to exhibit a unique distribution of miRNA and protein expression along the villus-crypt axis for intestinal homeostasis. This homeostatic expression, however, is vulnerable to dynamic perturbation due to an altered expression of CD98, a protein implicated in the pathogenesis of IBD both experimentally and clinically. We have previously shown a dysregulation of miRNAs and protein expression levels along the ileal villus-crypt axis with a knockout of a single gene *PepT1*, which codes for a peptide transporter in IEC, contributing to the molecular changes associated with cell death and proliferation^32^. The molecular and functional impact of a gene deletion, or gene overexpression as in current study, is highlighted by the comprehensive undertake in identifying miRNAs and proteins selectively affected by the target genes, potentially contributing to the changes in IE function^7,33,34^ and inflammation^12,13,32,35,36^ in experimental colitis.

Emergence of computational biology and its evolvement into bioinformatics with user-friendly interfaces and the access to public databases have enabled non-computational researchers to process biological information obtained from high-throughput approaches^30^. Here, we have applied bioinformatics as a research tool to better understand and exploit data obtained from the miRNA PCR panel and proteomics analyses. This approach compares in parallel a differentially expressed miRNA and protein signatures in the same tissue obtained from the mouse ileal epithelium. We then elucidated the interactions among the dysregulated proteins targeted by the differentially expressed miRNAs with potential consequences that may relate to the functional changes characterized in experimental colitis and CAC.

CD98 (SLC3A or 4F2) is a regulator of β1-integrin in the IE^37,38^ and is covalently linked to one of the light chains of LAT, rendering it as a facilitator of cellular processes involved in nutrition and metabolism^1,37,39,40^. Therefore, a consequential biological outcome of altering CD98 expression on the cell surface is potentially pervasive, predisposing the cell for changes deviant from the homeostatic regulation. Here we show that an alteration to CD98 expression in IECs modulates miRNA expression in the intestinal villus-crypt axis that may potentiate widespread post-transcriptional changes affecting various networks of genes and target proteins.

A miRNA may target a single or multiple protein-encoding genes while a downstream product of mRNA may be the consequence of being modified by a single or multiple miRNAs^21,41,42^. Our study demonstrates miRNAs having single or multiple putative targets dysregulated by CD98 overexpression. A gene coding for elongation factor-2, *Eef2*, is targeted by five miRNAs (miR-322-5p, -497-5p, -709, -326-3p, -484), of which three also targeted other genes, whereas a gene coding for carbamoyl-phosphate synthetase-1, *Cps1*, is the sole target of miR-151-5p that has no influence on other dysregulated proteins. In addition, our present study has observed a region-specific (villus or crypt) regulation of a protein-coding gene by varying sets of miRNAs. In WT villus, for example, downregulated expression of miR-455-3p and miR-326-3p influenced the expression of Atic (Bi-functional purine biosynthesis protein). However, a decreased expression of the same genomic target in CD98 overexpressing villus is regulated by miR-30e-3p. Such phenomena are evident with other differentially expressed miRNAs and their targets in this study. These results suggest that such control of genomic targets by miRNAs are incongruent and that the regulation may be unique to the function or the characteristics of the cell/tissue, in addition to the disease pathology.

Moreover, our study highlights the complexity of post-transcriptional regulation by miRNAs, as we have demonstrated here that merely decreased or increased miRNA expression levels do not translate directly to up or downregulated target protein expression levels, respectively. The mechanisms by which miRNAs facilitate mRNA translation activities are multifaceted, and the process encompasses destabilization and degradation of the genomic target through various mechanisms, where in certain conditions stimulate protein synthesis^43^. Indeed, an examination of the changes in the expression levels of miRNAs and putative targets in this study did not always show an inverse association (cross-examination of Figs 3 and 5, Supplementary Fig. S6 and Table 3). We speculate such dynamic modification enabled by the miRNAs to contribute to the unique gradient profile of target proteins we were able to establish for the ileal villus-crypt axis for the maintenance of homeostasis and CD98 gene modification in WT and Tg. Likewise, not all differentially expressed putative genomic targets in our study were targeted by variably expressed miRNAs, suggesting susceptibility to distribution perturbation via other means, contributing considerably to a certain aspect of disease development, independent of altered miRNA expression.

We have taken our analysis further to determine potential presence of interactions among the significantly expressed proteins in which their protein-coding genes are influenced by miRNAs. Moreover, these interacting proteins were analyzed for its functional implication that may provide additional insights pertaining to the disease or its mechanism. Herein, we show that the biological processes mostly, but not exclusively, affected by the interacting proteins are metabolism, cell-cell adhesion and junction, protein processing in ER, ECM, and exosome. Perhaps it isn’t implausible to speculate the dysregulation of CD98 expression to “precondition” the cell to exhibit an exacerbation of disease pathology in presence of an aggravator by altering the villus-crypt axis miRNA and protein expression profile. We have previously reported greater CD98 expression in IEC to have an insignificant effect on the basal miRNA expression profile in colon, while profoundly influencing the signature of some of the colonic miRNAs in exposure to DSS^25^. In presence of DSS-induced colitis, greater CD98 expression lead to a more profound disruption to the intestinal epithelial barrier function perpetuating the inflammatory cell infiltration and the exacerbation of colitis and CAC in the affected mice^13^. Implication of CD98 in certain aspects of tumor development and cell transformation is also evident due to its intimate coupling to β-integrin. By increasing the activation of focal adhesion kinase and stimulating its downstream pathways^13,40,44,45^ and encouraging cellular interaction with the microenvironment and aggregation through adhesion signaling^46^, CD98 support cell survival and proliferation. In a separate study, increased CD98 expression was identified in the intestinal adenomas of a Apc^Min/+^ (model of spontaneous adenoma development), in which the incidence of intestinal tumors were also increased when CD98 were overexpressed in their IE^15^. Clinically, positive CD98 staining and increased expression of CD98 in various cancer specimens were correlated with late cancer stages, cancer malignancies and metastasis, and poor patient survival outcomes^47–49^. Indeed, CD98 heavy chain augmentation in cells *in vitro* showed failure to initiate cell cycle arrest and apoptosis under nutrient-deprived condition^50^ and promotion of tumorigenic characteristics^51^.

Our results are only applicable to the CD98-overexpressing Tg model and the analyses used in the study despite our effort to compare morphologically similar fractions of ileal cells. The study does not assume the role or lack thereof the impact CD98 overexpression primarily in villus has on the stem cell niche in crypt and its subsequent effect during differentiation. Unfortunately, interpretation of our results is also limited by the absence of accompanying mRNA gene expression information. Nevertheless, our comprehensive approach elucidates a deviation in the miRNA-protein axis in ileal villus and crypt due to an altered expression of a surface protein associated with intestinal inflammation; such breadth of information is difficult to ascertain by the means of simple assays for a single target protein and/or miRNA(s).

In summary, an overexpression of CD98 in IEC potentiates the modulation of miRNA expression along the axis, thereby creating a unique gene and protein expression profile apart from what is required for the maintenance of epithelial homeostasis. We have applied the use of various bioinformatics to determine differential dysregulation of gene network and their putative target proteins in an attempt to provide insight to the biological processes affected by a single gene modification in the IEC as a prelude to further understand the role of CD98 in intestinal inflammation.

## Materials and Methods

### Animals

Female FVB wild-type (WT) and hCD98 transgenic (Tg) mice of 6–9 weeks of age were used for the following experiments. Transgenic CD98 mice were previously characterized^13^. DNA extraction from the tail or ear punch for genotyping was done using RED Extract (Sigma XNAT) following the manufacturer’s protocol. Primers used to determine the Tg mice were as follows: Villin Forward 5′-GGCTGTGATAGCACACAGGA-3′; hCD98 Reverse: 5′-CCTTGGACAGGCCCGTGAACTTA-3′. DNA amplification of the target gene was obtained under the following condition: 94 °C for 2 minutes, 94 °C for 30 seconds, 62 °C for 30 seconds, 72 °C for 40 seconds and 72 °C for 10 minutes for a total of 40 cycles. All animals were housed in the animal facility under 12:12 (light:dark) hour cycle where food and water were provided *ad libitum*. All procedures and use of mice were performed in accordance with and approved by the Georgia State University Institutional Animal Care and Use Committee (Atlanta, GA).

### Isolation of crypt and villus from the small intestine

Epithelium isolation procedures have been adopted and modified from the previous method^26^ and illustrated in Fig. 1a. Briefly, ileum of the small intestine (SI) from WT (n = 6) and Tg (n = 6) was removed and cleaned with cold HBSS. Luminal surface was exposed, cut into smaller pieces, and further washed in 150 ml cold HBSS + 0.5 mM DTT for 10 minutes at 4 °C with constant stirring at 200 rpm. Detached epithelium in the solution was decanted and collected as *Fraction 1* (Step 1). The remaining tissue was then washed in 100-ml cold chelating buffer for 40 min at 4 °C with constant stirring at 200 rpm. Detached epithelium in this solution was decanted and collected as *Fraction 2* (Step 2). The remaining tissue was then transferred to a fresh 20-ml chelating buffer and manually inverted 60 times. Detached epithelium in this solution was decanted and collected in a new 50-ml conical tube. The manual invert was repeated three times and collectively assigned as *Fraction* 3 (Step 3). The washing of the remaining tissue and decanting and collecting the epithelium (Steps 2–3) were repeated, with 20 minutes of stirring, four times for the subsequent fractions (*Fractions* 4, 5, 6, 7, 8, 9, 10, 11). Lastly, after the collection of *Fraction* 11, the remaining tissues were added to a fresh 20-ml cold chelating buffer and shaken vigorously for 1 minute, where the epithelial cells in the solution were collected as *Fraction* 12 (Step 4). This step was repeated to yield *Fraction* 13. All collected fractions were centrifuged at 2500 × *g* for 5 minutes to yield pellets of epithelial cells. The pellets were then washed and re-suspended in HBSS. Confirmation of crypt and villus were made through microscopy using Nikon Eclipse TS100 (4X and 10X magnification) and RT-PCR. Samples were stored in −80 °C until further use.

### RNA isolation and real-time PCR

Total RNA were extracted from all of the tissue fractions using miRCURY RNA Isolation Kit (Exiqon, Woburn, MA) or Purelink Mini RNA Kit (Invitrogen) according to the manufacturer protocol for purifying RNA from animal tissues. First-strand cDNA synthesis and real-time PCR for the total RNA were performed using Maxima First-strand cDNA synthesis and Maxima SYBR Green/ROX qPCR Master Mix kit (Thermo Scientific), respectively.

cDNA synthesis and real-time PCR for miRNA were performed using NCode miRNA First-strand cDNA synthesis (#MIRC-50, Sigma) and NCode SYBR GreenER qPCR SuperMix Universal (MIRQER-100). mRNA expression levels were calculated using ΔCt methods.

### miRNA expression analysis

Total RNA samples (50 ng/µl) from WT and Tg villus (n = 4 for each genotype) and crypt (n = 4 for each genotype) underwent PCR reactions conducted by Exiqon Services (Exiqon Services, Vedvack, Denmark).

The samples underwent quality control using several spike-ins (UniSp6, UniSp3) provided by Exiqon Services. Normalization of the data was also performed by applying the average of the assays detected in all samples (n = 20) as a suitable method for stable normalization^52^. The method included 134 assays and used the Normfinder software^53^. The equation used to measure normalized Ct value is the following:

$$Normalized\,Ct=average\,Ct(n=20)-assay\,Ct\,(sample)$$

#### miRNA qRT-PCR

Reverse transcription was done using miRCURY LNA^TM^ Universal RT microRNA PCR, Polyadenylation and cDNA synthesis kit (Exiqon). cDNA was diluted 100x and assayed in 10 µl PCR where each microRNA was assayed once by qPCR on the microRNA Ready-to-Use PCR, Mouse&Rat panel I + II using ExiLENT SYBR® Green master mix. Amplification was performed in a LightCycler® 480 RealTime PCR System (Roche) in 384-well plates. The amplification curves were analyzed using the Roche LC software, both for determination of Ct (with the second derivative method) and for melting curve analysis.

#### miRNA data analysis and potential target prediction

Assays were inspected and checked for melting curves and Tm, using the algorithm designated for the calculation of amplification efficiency. Moreover, only the assays detected with Ct’s below 37 and 5 Ct’s less than the negative control were included in the data analysis. Data deviant from these criteria were omitted from further analysis. Ct was calculated as the 2^nd^ derivative. For the normalization of the data, average of assays detected in all samples (average – assay Ct) determined by NormFinder was applied. Predicted potential target genes of identified miRNAs were determined using miRSearch V3.0 algorithms (http://www.exiqon.com/mirsearch; Exiqon, Woburn, MA).

### Proteome analysis and protein expression

Total protein from the tissue samples villus (n = 4 for each genotype) and crypt (n = 4 for each genotype) of WT and Tg was isolated and pooled. Concentrations were determined using ToPI-DIGE (Differential In-Gel Electrophoresis) kit and ToPA kit and further processed using ITSIPrep ToPrep Kit (ITSI Biosciences, LLC, Johnstown, PA).

#### Protein separations and image analysis

Fluorescent dyes Cy3 or Cy5 in 200 picomole were used to label pooled samples of WT villus, WT crypt, Tg villus or Tg crypt. Universal internal control (S) was achieved by pooling the same amount of proteins (50 µg) from all samples which were labeled with dye Cy2 and added to each gel as a reference for quantitative comparisons of all samples. Labeled samples were combined as the following for two gels: Gel 1–WT Villus (Cy3), WT Crypt (Cy5), S (Cy2); Gel 2 – Tg villus (Cy3), Tg Crypt (Cy5, S (Cy2). For the 1^st^ dimension separation, samples each labeled with dye Cy2, Cy3, or C5, were mixed and loaded to a single 24 cm IEF strip, pH 3–10 NL. The strips were then rehydrated for 12 hours at 30 volts, followed by 65,000 volt hours of focus. For 2^nd^ dimension separation, focused strips were loaded onto a 24 cm × 20 cm, 12.5% SDS-PAGE gel and ran for 4 hours. Thereafter, the gels were exposed to three wavelengths and scanned with a Typhoon DIGE-Enabled Digital Imager to capture the emitted signals from the dyes. Image analysis was performed using the Differential In-Gel Analysis (DIA) and Biological Variation Analysis (BVA) modules of DeCyder software (GE Healthcare). Spot volumes from 2D-DIGE gels were compared as follows: 1) WT villus vs. WT crypt; 2) Tg villus vs. Tg crypt; 3) Tg villus vs. WT villus; 4) Tg crypt vs. WT crypt. Spot volume ratio greater than 2 fold was considered differentially expressed, thus included as a candidate protein. Accession number obtained from FASTA databases for mouse from Uniprot. Gene symbol and Protein name from Uniprot and MGI databases. Score, number of unique peptides, MW [kDA], and Protein pI are the parameters obtained from peptide digest result for the identified proteins. Genes are of *Mus musculus*.

#### Gel spot picking and liquid chromatography-tandem mass spectrometry (LC-MS/MS) analysis

2D-DIGE gels were stained with Lava Purple Gel stain to increase spot map accuracy. In total, 32 spots were chosen to undergo tryptic digestion by spot handling robots. Spots were then de-stained and digested overnight. Digested extracts were then re-suspended and desalted with a Zip tip and dried again in a Speed Vac. Samples underwent another resuspension in 2% acentonitrile/0.1% formic acid and subjected to nanospray column mass spec analysis using LTQ XL mass spectrometer (Thermo Scientific). Digested peptide sequences were searched against FASTA databases for Mouse from Uniprot using Proteome Discoverer 1.4 (Thermo Scientific) and the SEQUEST algorithm.

### miRNA target prediction

Predicted miRNA-gene targets were investigated using miRWalk 2.0 (http://zmf.umm.uni-heidelberg.de/apps/zmf/mirwalk2/, Germany). A family of six *let-7* miRNAs was excluded from the target assessment of 119 miRNAs. Potential genomic targets of 112 miRNAs were matched against 62 genes identified through Uniprot that are responsible for encoding the putative proteins found to be differentially expressed from the proteomics analysis.

### Functional annotation clustering and protein interaction

Investigation of protein-protein interaction was performed using STRING v10.0 (Search Tool for the Retrieval of Interacting Genes/Proteins, http://string-db.org). Degrees of interaction amongst the 29 proteins (in *Mus musculus*) were identified and visualized using the interaction network view. The Edge Confidence indicates the confidence of functional association or interaction based on the evidence from experimental and knowledge-based databases used by STRING. Nodes (colored circle) represent proteins where large size indicates an evidence of known or predicted 3-dimensional protein structure.

Database for Annotation, Visualization and Integrated Discovery (DAVID 6.8, https://david.ncifcrf.gov/summary.jsp) bioinformatics annotation tool bioinformatics analysis tool was used to generate Functional Annotation Clustering report for the proteins shown to interact with each other from the STRING analysis. Proteins with enrichment scores ≥1.0 were selected, and their “GOterm_direct” categories were used to search for GO (Gene Ontology) terms. The strength of enrichment for the annotation categories were identified with p-value (EASE score p-value; modified Fisher’s Exact Test 3), which reflect the threshold probability of biological significance. Classification stringency for generating annotation clustering was set at “medium.”, in which GO terms in Enriched Functional Group Gene Annotation/Function with p-values ≥ 0.05 were omitted from the analysis.

### Data analysis

The threshold for significance was set at 0.05. For the miRNA data analysis, two-sample t-test was used to determine the levels of miRNAs differentially expressed between the groups. For the mRNA expression data, two-sided Paired t-test was used.

## Electronic supplementary material

Supplementary Information

## Data Availability

The datasets generated or analyzed during the present study are available from the corresponding author on reasonable request.
