# Supplementary material for: Overexpression of CD98 in intestinal epithelium dysregulates miRNAs and their targeted proteins along the ileal villus-crypt axis
Source: Sci Rep. 2018 Nov 1;8:16220. doi: 10.1038/s41598-018-34474-9 (PMC6212412; doi:10.1038/s41598-018-34474-9)
Supplement: Supplementary file 1 — Supplementary Information [file 41598_2018_34474_MOESM1_ESM.pdf]

Supplementary Information

Overexpression of CD98 in intestinal epithelium dysregulates miRNAs and their targeted proteins along the ileal villus-crypt axis

Authors

Moon K. Han[1,*], Mark Baker[1], Yuchen Zhang[1], Chunhua Yang[1], Mingzhen Zhang[1], Pallavi Garg[1], Emilie Viennois[1], and Didier Merlin[1, 2]

Affiliations

[1]Institute for Biomedical Sciences, Center for Diagnostics and Therapeutics, Digestive Disease Research Group, Georgia State University, Atlanta, 30303, USA

[2]Atlanta Veterans Affairs Medical Center, Decatur, 30033, USA

*Corresponding author:

Moon K. Han, MS

Georgia State University

Petit Science Center, Rm 757

100 Piedmont Ave SE

Atlanta, GA 30303

Email: mkim15@gsu.edu

Supplementary Figure S1.1. Overexpression of CD98 in IE yields irregularity in the expression of abundance in proteins in the villus and crypt. 2D-DIGE gel image was analyzed using Biological Variation Analysis modules of DeCyder software. Only the spots identified with a volume ratio difference of ≥ 2.0 between any of the comparisons (**a** vs. **b**; **c** vs. **d**; **a** vs. **c**; **b** vs. **d**) were considered differentially expressed or abundant. Gel images are cropped but spots present in each panel display are all inclusive and no spots were left discarded. Uncropped, and untouched original gel images of the corresponding gels are present in **Supplementary Figure S1.2**.

Supplementary Figure S1.2. 2D-DIGE gel image was analyzed using Biological Variation Analysis modules of DeCyder software. Displayed images are the original uncropped and untouched images of the corresponding gels in **Supplementary Figure S1.1.**

Supplementary Figure S2. CD98 overexpression selectively alters the expression levels of proteins common to villus-crypt axis of ileal epithelium. 2D-DIGE gel image was analyzed using Biological Variation Analysis modules of DeCyder software. Of the 318 spots identified, 20 spots with volume ratio ≥ 2.0 common to axial comparisons (**a** vs. **b** for WT villus-crypt axis; **c** vs. **d** for Tg villus-crypt axis) were chosen for further analysis. The images are uncropped and correspond to the original image with minor exposure adjustments under the same setting for all images for better visualization of the spots.

Supplementary Figure S3. CD98 overexpression disturbs protein expression distribution that is unique to villus and crypt, contributing to the differential villus-crypt axial protein expression profile. 2D-DIGE gel image was analyzed using Biological Variation Analysis modules of DeCyder software. Of the 318 spots, 8 spots from the villus comparison (**a** vs. **c**) and 7 spots from the crypt comparison (**b** vs. **d**) were randomly chosen based on a fold change and location on the gel. The images are uncropped and correspond to the original image with minor exposure adjustments under the same setting for all images for better visualization of the spots.

Supplementary Figure S4. Differentially expressed protein distribution along the axis is affected by CD98 overexpression. An overlay of differentially expressed proteins in the axis of WT and Tg has shown 9 proteins above and 24 proteins below the expression level that of the WT in the axis of Tg. Only one protein Capza1 (F-actin-capping protein subunit alpha-1) showed no change in the expression level along the axis due to CD98 overexpression (overlap of red circle and black triangle). Dotted lines indicate the threshold for 2-fold change. Y-axis is in Log_2 (Spot volume ratio) scale.

Supplementary Figure S5. miRNA distribution and variation profile is different in WT and CD98 Tg IECs. Top 50 miRNAs (normalized to ΔCt) with the largest variations for each group has been plotted for the analysis based on the expression profile to visualize variations in miRNA between the two genotypes using Principal Component Analysis (PCA; PC1: Largest component in variation; PC2: Second largest component in variation, **a**) as an overview. A differential clustering of miRNA expression can be seen based on the groups. There is a distinct separation of villus and crypt for each

genotypes but partial overlap in the clustering based on villus or crypt. The miRNA clustering tree of the heat-map diagram (**b**) is shown on left while sample clustering is shown on top. Clustering is performed on all samples and on the top 50 miRNAs with highest standard deviation. Each row represents one microRNA, and each column represents one sample. Color scale represents relative expression level about the mean of miRNAs across all samples.

Supplementary Figure S6. **Distribution of certain miRNAs were minimally influenced by CD98 overexpression.** Relative fold change ($2^{-\Delta\Delta CT}$) of similarly expressed miRNAs in villus-crypt axis of WT and CD98 Tg ileal IECs is shown. All miRNAs listed were found to be differentially expressed using a cutoff p-value of < 0.05. A total of 37 miRNAs were similarly distributed in both WT and Tg villus-crypt axis, of which 13 were downregulated (**a**, **b**) and 24 were upregulated (**c**, **d**) in villus compared to crypt. Y-axis is in Log_2 (Relative expression) scale.

Supplementary Table S1. **Number of 2D-DIGE gel spots with volume ratio greater than 2-fold difference for each comparison.**

Supplementary Table S2.1. Distinctive protein profile along the ileal villus-crypt axis is disrupted in CD98 overexpressing mice. Selection of top two putative proteins per spot (unless only one was identified) ranked by protein score and number of unique peptides has yielded 62 proteins including several that were identified in

repeats. Protein with highest score is shown in **Table 1** while repeats are shown in this table. Average fold change in axis refers to changes in villus compared to its respective crypt within same genotype. Average fold change between villus or crypt refers to changes between two different genotypes. (-) sign refers to downward fold change.

Supplementary Table S2.2. Distinctive protein profile along the ileal villus-crypt axis is disrupted in CD98 overexpressing mice. Selection of top two putative proteins per spot (unless only one was identified) ranked by protein score and number of unique peptides has yielded 62 proteins including several that were identified in repeats. Protein with highest score is shown in **Table 1** while repeats are shown in this table. Average fold change in axis refers to changes in villus compared to its respective crypt within same genotype. Average fold change between villus or crypt refers to changes between two different genotypes. (-) sign refers to downward fold change.

Supplementary Table S3. miRNAs levels in villus and crypt are differently expressed under the influence of CD98 overexpression compared to the WT mice.

Supplementary Table S4. Enriched functional clustering of interactive putative genes/proteins targeted by miRNAs in the villus-crypt axis of WT. Functional annotations of gene clusters were determined by DAVID Functional Annotation Bioinformatics Microarray Analysis. Count: Number of putative genes from the list in the cluster contributing to the enriched function; Genes: List of genes contributing to the functional group annotation cluster.

Supplementary Table S5. Enriched functional clustering of interactive putative genes/proteins targeted by miRNAs in the villus-crypt axis of Tg. Functional annotations of gene clusters were determined by DAVID Functional Annotation Bioinformatics Microarray Analysis. Count: Number of putative genes from the list in the cluster contributing to the enriched function; Genes: List of genes contributing to the functional group annotation cluster.

Supplementary Table S6. Enriched functional clustering of interactive putative genes/proteins targeted by similarly expressed miRNAs in the villus-crypt axis of WT and Tg. Functional annotations of gene clusters were determined by DAVID Functional Annotation Bioinformatics Microarray Analysis. Count: Number of putative genes from the list in the cluster contributing to the enriched function; Genes: List of genes contributing to the functional group annotation cluster.

Supplementary Figure S 1.1

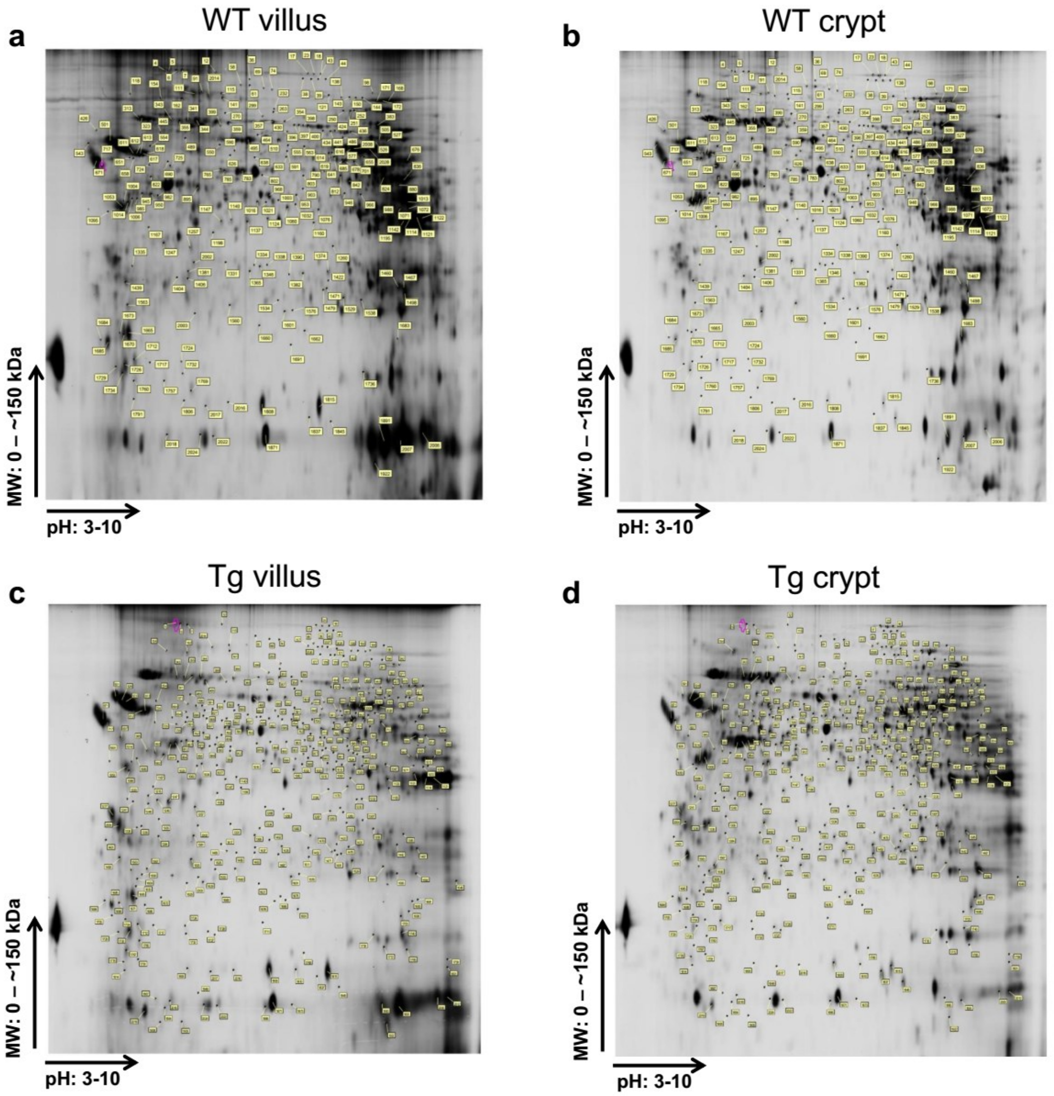

Supplementary Figure S 1.2

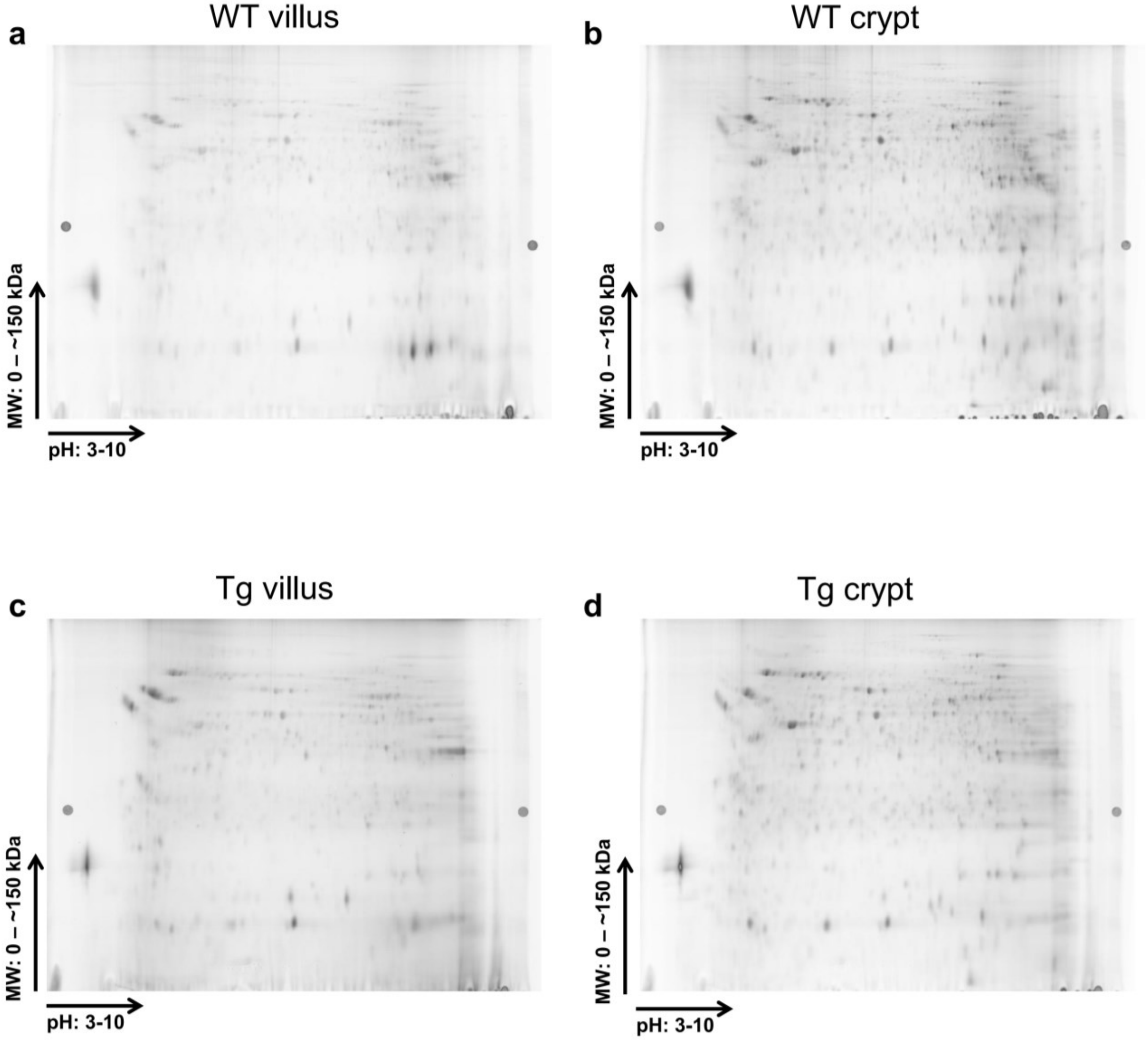

Supplementary Figure S 2

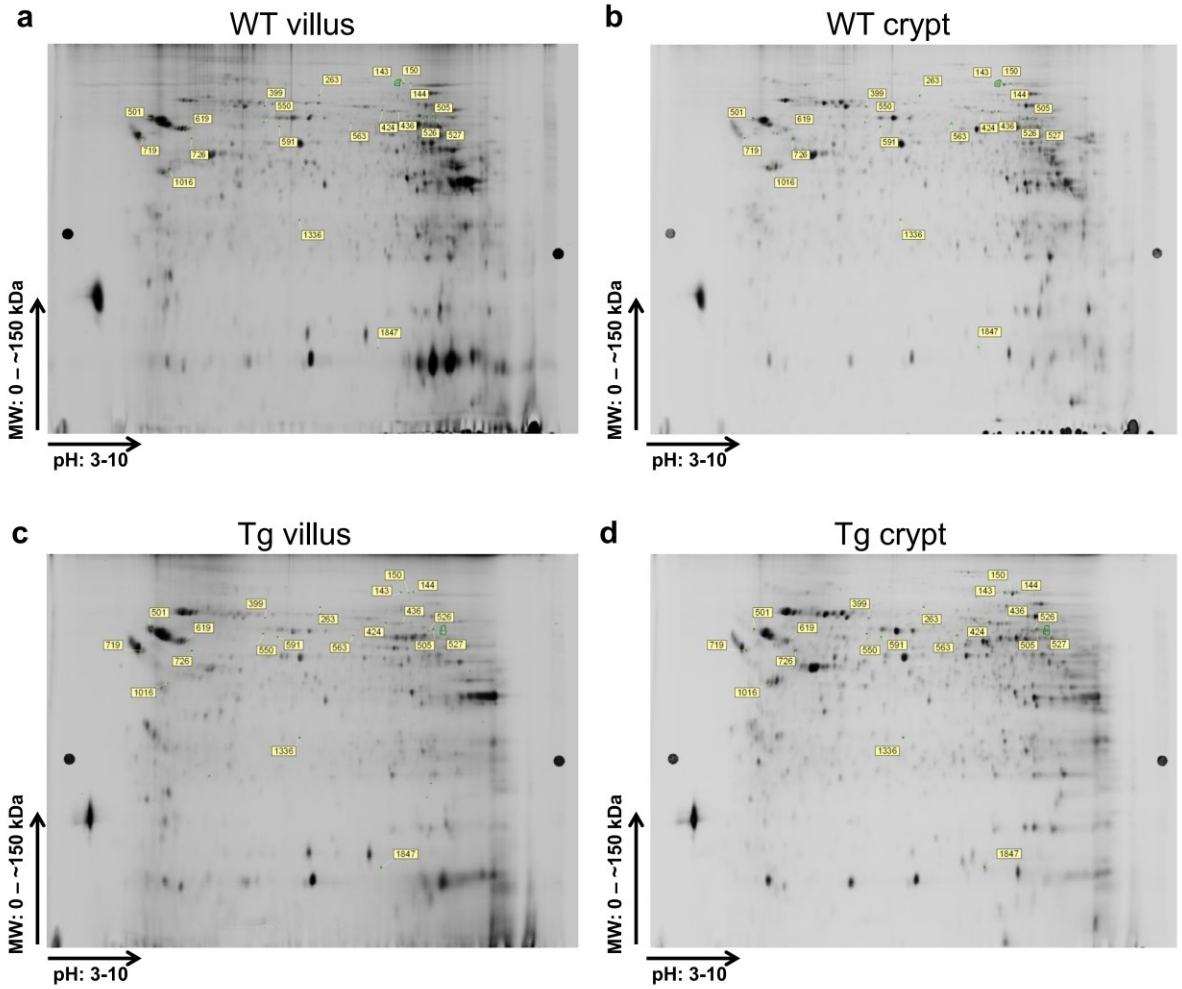

Supplementary Figure S 3

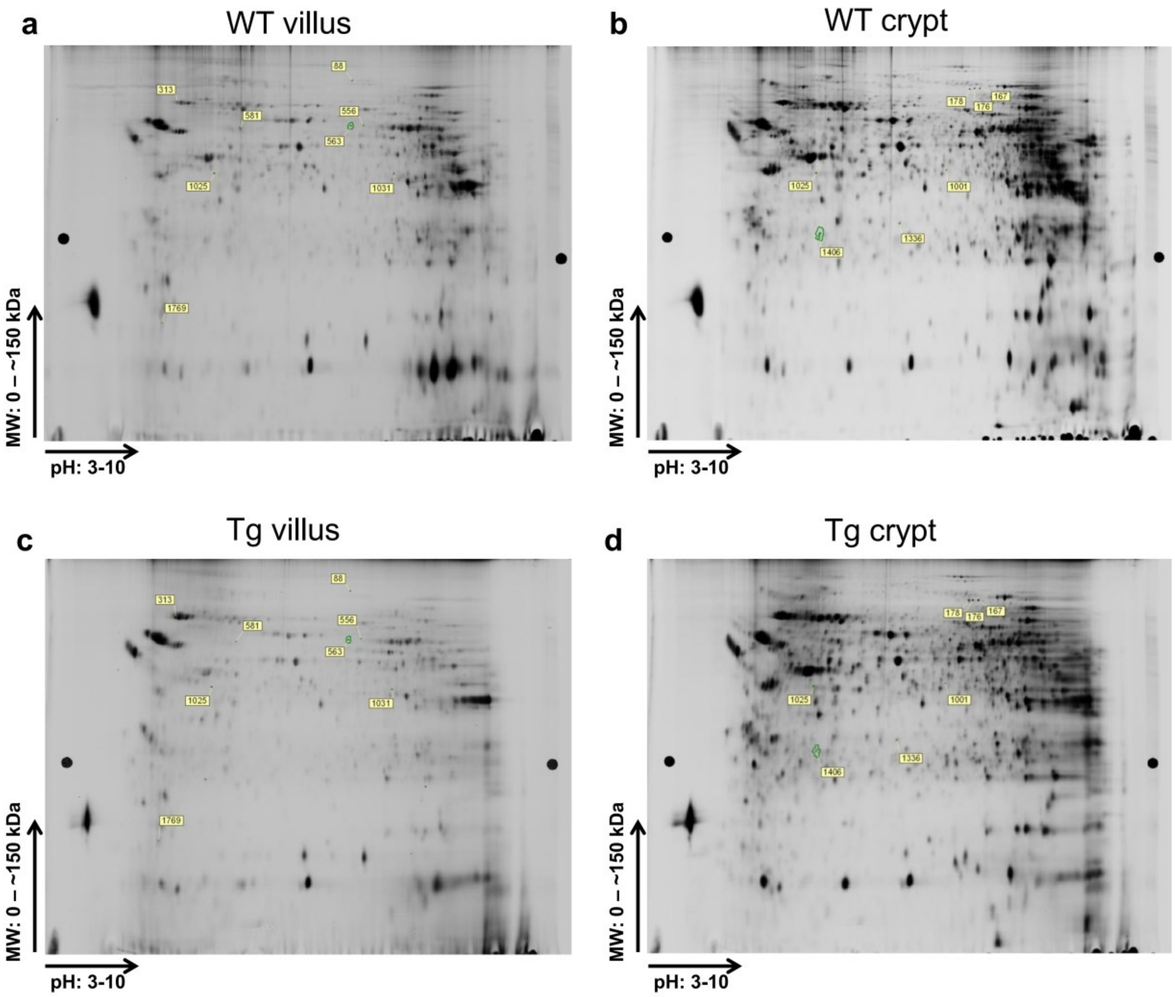

Supplementary Figure S 4

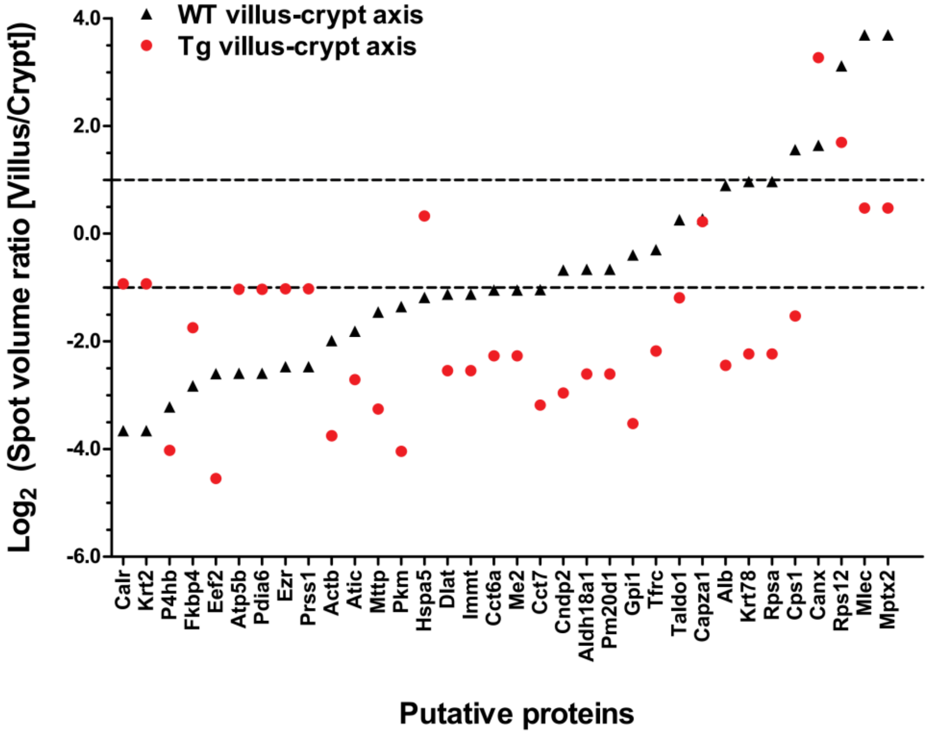

Supplementary Figure S 5

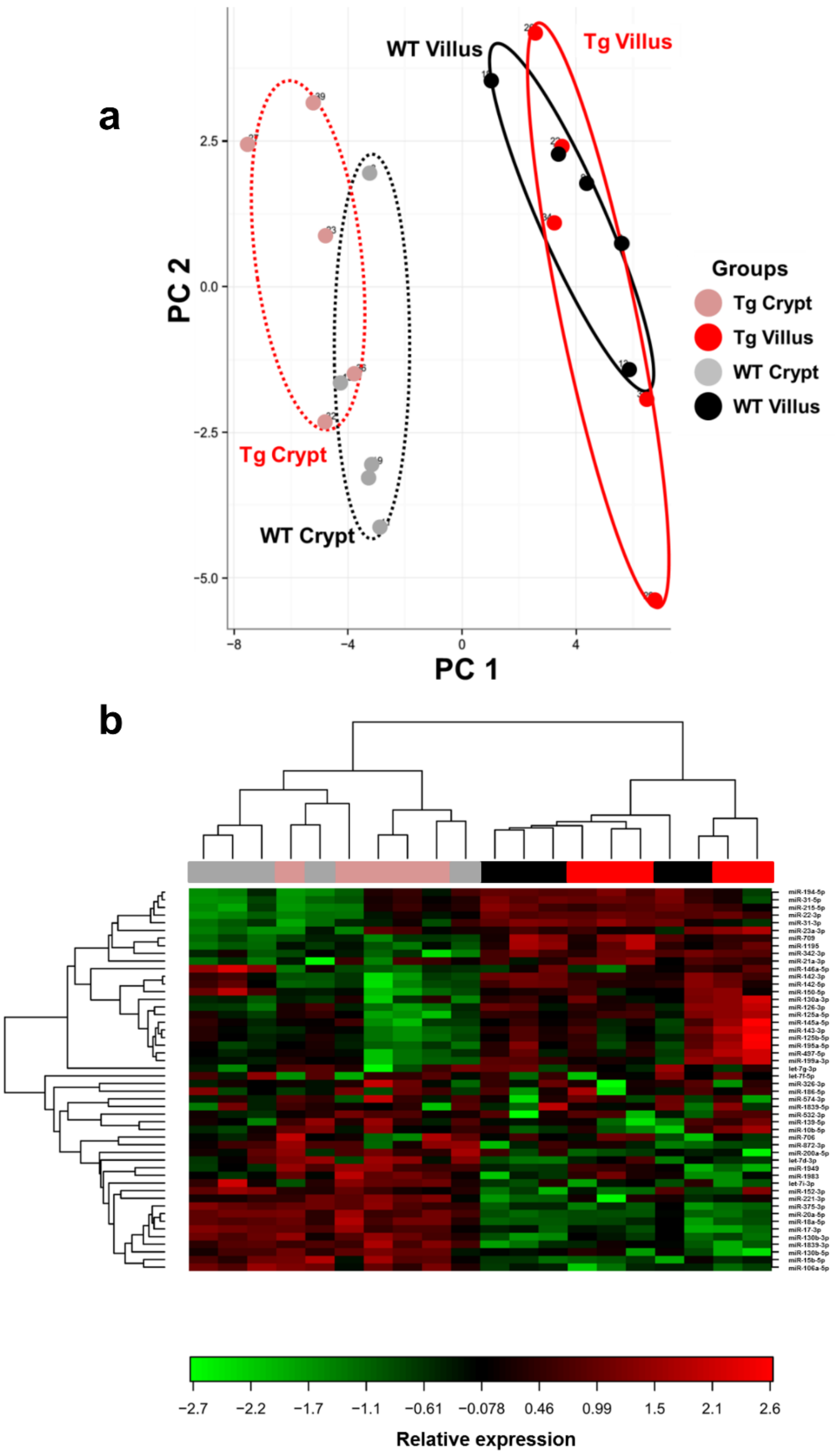

Supplementary Figure S 6

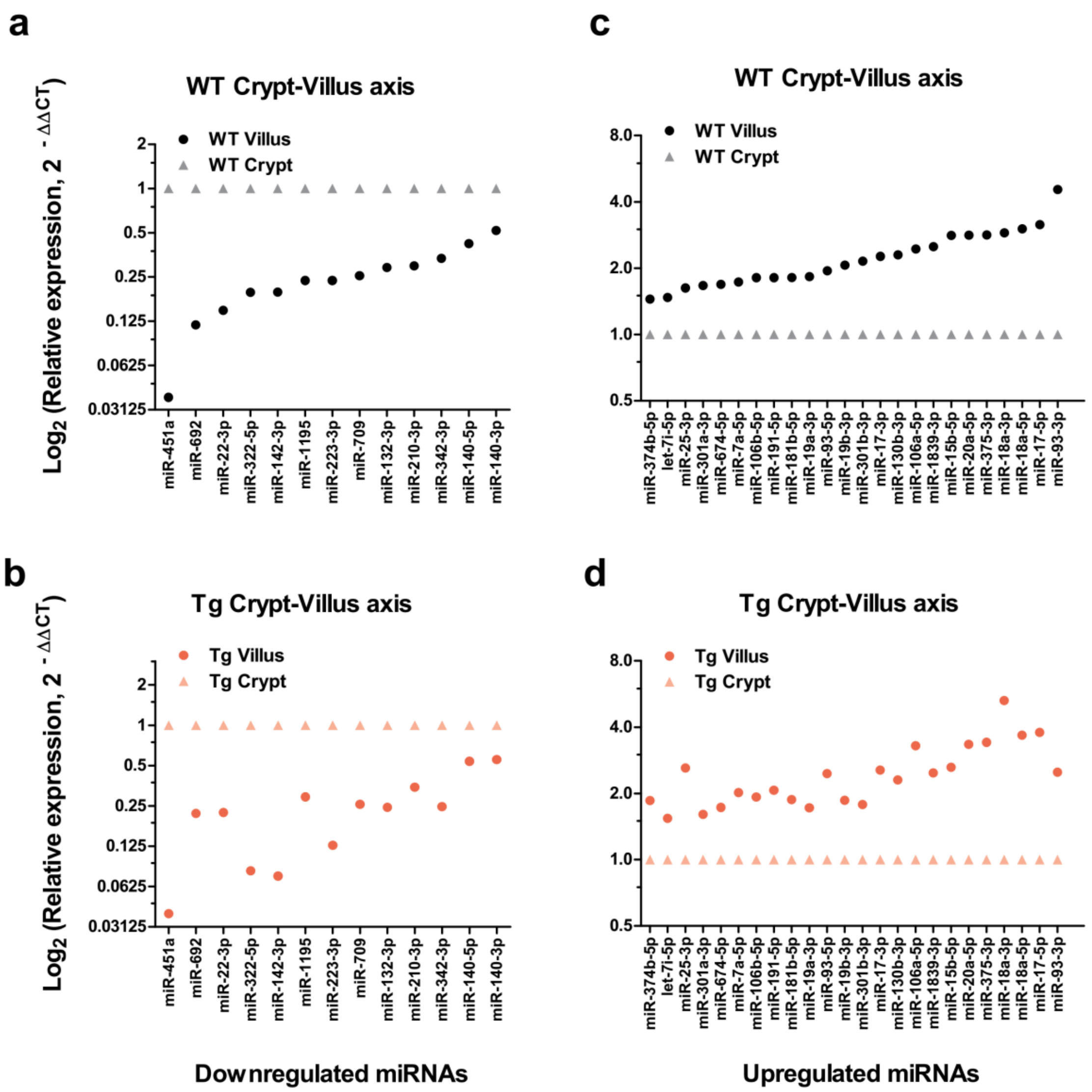

Supplementary Table S1. Number of 2D-DIGE gel spots

Number of 2D-DIGE Gel Spots	WT Villus-Crypt Axis[a]	Tg Villus-Crypt Axis[b]	Between Villus[c] (Tg : WT)	Between Crypt[d] (Tg : WT)
Volume ratio ≥ 2.0	115	140	30	39
Volume ratio ≤ 2.0	106	226	63	30
Total	221	366	93	69
Selected for analysis	20		8	7

[a]Expression in WT villus relative to WT crypt
[b]Expression in Tg villus relative to Tg crypt
[c]Expression in Tg villus relative to WT villus
[d]Expression in Tg crypt relative to WT crypt

Supplementary Table S2.1. Distinctive protein profile along the ileal villus-crypt axis

Spot No.[a]	Accession ID[b]	Protein Name	Gene	Score[c]	No. of Unique Peptides[d]	MW [kDa]	Protein pI[e]	Relative expression Log$_2$ (Spot Volume Ratio)			
								WT Villus-Crypt Axis	Tg Villus-Crypt Axis	Between Villus (Tg : WT)	Between Crypt (Tg : WT)
424	P07724	Serum albumin	Alb	13.32	1	68.6	6.07	-1.82	-2.71	-0.55	0.35
1031	Q9CWJ9	Bifunctional purine biosynthesis protein PURH	Atic	15.09	1	64.2	6.76	0.25	-1.19	-2.05	-0.61
619	P56480	ATP synthase subunit beta, mitochondrial	Atp5b	78.96	8	56.3	5.34	-3.22	-4.02	-1.80	-1.00
526	P56480	ATP synthase subunit beta, mitochondrial	Atp5b	20.4	3	56.3	5.34	-1.36	-4.04	-2.15	0.53
550	P80313	T-complex protein 1 subunit eta	Cct7	50.22	5	59.6	7.84	-2.83	-1.75	0.99	-0.09
1025	Q6KAT3	Cytosolic non-specific dipeptidase	Cndp2	14.16	1	44.3	5.38	0.27	0.22	-2.15	-2.10
143	Q6P9L9	Eef2 protein (Fragment)	Eef2	96.21	8	93.5	6.83	-1.99	-3.75	-1.65	0.11
313	Q6P9L9	Eef2 protein (Fragment)	Eef2	74.83	4	93.5	6.83	-1.19	0.33	2.06	0.54
144	Q6P9L9	Eef2 protein (Fragment)	Eef2	39.39	4	93.5	6.83	-1.46	-3.26	-1.52	0.28
1847	P58252	Elongation factor 2	Eef2	20.49	4	95.3	6.83	3.12	1.69	-0.99	0.43
178	Q6P9L9	Eef2 protein (Fragment)	Eef2	13.67	3	93.5	6.83	-0.30	-2.18	0.07	1.95
167	Q6P9L9	Eef2 protein (Fragment)	Eef2	6.79	2	93.5	6.83	1.56	-1.53	-0.63	2.46
1769	Q61509	Elongation factor 2 (Fragment)	Eef2	4.55	1	29.9	6.64	1.47	-0.11	-2.81	-1.23
591	B2RTP7	Krt2 protein	Krt2	10.28	1	70.9	8.06	-1.53	-2.57	-0.95	0.10
1016	B2RTP7	Krt2 protein	Krt2	8.83	1	70.9	8.06	1.81	3.23	0.34	-1.07

[a]Spot Number: Spot identity in gel

[b]Accession ID: Unique identifier of the protein based on FASTA database

[c]Score: Total score of the protein based on individual peptides

[d]Unique peptides: Number of peptide sequence unique to the protein group

[e]Protein pI: Theoretical isoelectric point based on protein molecules

Supplementary Table S2.2. Distinctive protein profile along the ileal villus-crypt axis

Spot No.[a]	Accession ID[b]	Protein Name	Gene	Score[c]	No. of Unique Peptides[d]	MW [kDa]	Protein pI[e]	Relative expression Log_2 (Spot Volume Ratio)			
								WT Villus-Crypt Axis	Tg Villus-Crypt Axis	Between Villus (Tg : WT)	Between Crypt (Tg : WT)
176	A1L0X5	Krt78 protein (Fragment)	Krt78	16.25	1	54.7	6.3	0.89	-2.45	-1.67	1.67
1001	A1L0X5	Krt78 protein (Fragment)	Krt78	12.99	1	54.7	6.3	1.13	-1.19	-0.39	1.94
505	Q99KE1	Malic enzyme 2, mitochondrial NAD-dependent	Me2	28.3	4	65.8	7.61	-1.62	-3.42	-1.90	-0.10
501	Q3UDR2	Prolyl 4-hydroxylase, beta polypeptide	P4hb	71.78	7	56.6	4.89	1.64	3.27	1.17	-0.45
1016	Q3TT76	Prolyl 4-hydroxylase, beta polypeptide	P4hb	21.24	2	43	5.14	1.81	3.23	0.34	-1.07
505	P52480	Pyruvate kinase PKM	Pkm	53.98	6	57.8	7.47	-1.62	-3.42	-1.90	-0.10
527	P52480	Pyruvate kinase PKM	Pkm	27.16	4	57.8	7.47	-1.04	-3.18	-1.98	0.17
581	P52480	Pyruvate kinase PKM	Pkm	19.32	3	57.8	7.47	-0.68	-2.96	-2.32	-0.04
563	Q8C165	Probable carboxypeptidase PM20D1	Pm20d1	18.18	3	55.6	6.43	-2.26	-5.13	-2.86	0.01
563	Q9Z1R9	MCG124046 (Protease, Serine, 1)	Prss1	16.74	1	26.1	4.94	-2.26	-5.13	-2.86	0.01
1001	Q9Z1R9	MCG124046 (Protease, Serine, 1)	Prss1	13.3	1	26.1	4.94	1.13	-1.19	-0.39	1.94
88	Q9Z1R9	MCG124046 (Protease, Serine, 1)	Prss1	12.94	1	26.1	4.94	-0.40	-3.53	-3.32	-0.18
150	Q9Z1R9	MCG124046 (Protease, Serine, 1)	Prss1	12.66	1	26.1	4.94	-2.60	-4.55	-1.74	0.20

[a]Spot Number: Spot identity in gel

[b]Accession ID: Unique identifier of the protein based on FASTA database

[c]Score: Total score of the protein based on individual peptides

[d]Unique peptides: Number of peptide sequence unique to the protein group

[e]Protein pI: Theoretical isoelectric point based on protein molecules

Supplementary Table S3. Differently expressed miRNAs in Tg compared to WT

Tg compared to WT	Villus comparison		Crypt comparison	
**Upregulated miRNAs**	let-7c-5p<br>let-7a-5p<br>miR-25-3p<br>miR-30e-5p<br>miR-215-5p	miR-140-5p<br>miR-185-5p<br>miR-26b-5p<br>miR-22-5p	let-7a-1-3p<br>miR-200a-3p<br>miR-148a-3p<br>miR-125b-5p<br>miR-99a-5p<br>miR-146a-5p<br>miR-133a-3p	miR-181c-5p<br>miR-142-5p<br>miR-142-3p<br>miR-1a-3p<br>miR-150-5p
**Downregulated miRNAs**	miR-124-3p<br>miR-291b-5p<br>miR-29b-1-5p<br>miR-193b-3p<br>miR0466f-3p<br>miR-1198-5p<br>miR-134-5p<br>miR-149-5p<br>miR132-3p<br>miR-26a-1-3p<br>miR-30e-3p		let-7d-5p<br>let-7f-1-3p<br>miR-203-5p<br>miR-33-3p<br>miR-1983<br>miR-20a-3p<br>miR-148a-5p<br>miR-674-3p<br>miR-130b-5p<br>miR-134-5p<br>miR-1949<br>miR-194-2-3p<br>miR-423-3p<br>miR-324-5p	miR-324-5p<br>miR-7a-5p<br>miR-128-3p<br>miR-484<br>miR-182-5p<br>miR-200b-5p<br>miR-674-5p<br>miR-148b-3p<br>miR-151-5p<br>miR-103-3p<br>miR-191-5p<br>miR-21a-5p<br>miR-301-3p

Supplementary Table S4. Enriched functional clusters in WT villus-crypt axis

Annotation Cluster[a]	Enrichment Score	Term	Count	Genes
1	3.29	Cadherin binding, cell-cell adhesion	5	*Pkm, Atic, Capza1, Eef2, Hspa5, Fkbp4*
		Cell-cell adherens junction	5	
		Isopeptide bond	4	
2	2.46	Mitochondrion	8	*Pkm, Me2, Immt, Atic, Atp5b, Fkbp4, Dlat, Hspa5*
		Transit peptide	4	
3	2.32	Smooth endoplasmic reticulum	4	*Fkbp4, Pdia6, Cct6a, Calr, Canx, Hspa5, Actb*
		Protein folding	5	
		Chaperone	5	
		Uunfolded protein binding	4	
		Chaperone-mediated protein folding	3	
		Prevents secretion from ER	3	
		Glycoprotein binding	3	
		Protein processing in endoplasmic reticulum	4	
		Melanosome	3	
		Endoplasmic reticulum lumen	3	
		Protein complex	4	
		Endoplasmic reticulum	4	
4	2.22	Extracellular matrix	7	*Pkm, Actb, Immt, Atp5b, Capza1, Eef2, Cct6a, Hspa5, Calr, Canx, Fkbp4, Taldo1*
		Ubl conjugation	6	
		Protein binding	10	
		Methylation	5	
		Nucleotide-binding	6	
		ATP binding	6	
		Cytoplasm	8	
		Isopeptide bond	4	
5	1.9	Carbon metabolism	4	*Pkm, Me2, Taldo1, Dlat, Atic*
		Pyruvate metabolism	3	
		Biosynthesis of antibiotics	4	
		Metabolic process	4	
		Catalytic activity	4	

Annotation Cluster[b]	Enrichment Score	Term	Count	Genes
1	3.54	Extracellular exosome	7	*Rpsa, Fkbp4, Pdia6, Cct6a, Hspa5, Calr, Canx*
		Poly(A) RNA binding	5	
		Acetylation	6	
		Cytoplasm	6	
2	2.51	Smooth endoplasmic reticulum	4	*Pdia6, Hspa5, Calr, Canx, Cct6a*
		Unfolded protein binding	4	
		Protein processing in endoplasmic reticulum	4	
		Prevents secretion from ER	3	
		Glycoprotein binding	3	
		Melanosome	3	
		Endoplasmic reticulum lumen	3	
		Endoplasmic reticulum	4	

[a]Biological processes associated with putative targets of DOWNREGULATED miRNAs in WT villus-crypt axis

[b]Biological processes associated with putative targets of UPREGULATED miRNAs in WT villus-crypt axis

Supplementary Table S5. Enriched functional clusters in Tg villus-crypt axis

Annotation Cluster[a]	Enrichment Score	Term	Count	Genes
1	2.39	Protein folding	3	*P4hb, Fkbp4, Eef2, Calr*
		Chaperone	3	
		Poly(A) RNA binding	4	
2	2.27	Cytosol	6	*Actb, Cndp2, Fkbp4, Eef2, Hspa5, Gpi1, Calr*
		Ubl conjugation	5	
		Cytoplasm	6	
3	1.86	Extracellular matrix	4	*P4hb, Eef2, Hspa5, Calr, Actb*
		Prevents secretion from ER	3	
		Focal adhesion	4	
		Endoplasmic reticulum lumen	3	
		Protein processing in endoplasmic reticulum	3	
4	1.85	Extracellular matrix	4	*P4hb, Eef2, Hspa5, Calr, Actb, Fkbp4, Gpi1*
		Response to endoplasmic reticulum stress	3	
		Ubl conjugation	5	
		Myelin sheath	3	
5	1.46	Ubl conjugation	5	*Actb, Fkbp4, Eef2, Hspa5, Gpi1*

Annotation Cluster[b]	Enrichment Score	Term	Count	Genes
1	2.72	Metabolic process	6	*Pkm, Me2, Cndp2, Pm20d1, Dlat, Cps1*
		Carbon metabolism	4	
		Allosteric enzyme	3	
		Pyruvate metabolism	3	
		Transit peptide	3	
		Catalytic activity	3	
		Transit peptide_Mitochondrion	3	
		Mitochondrion	4	
2	2.49	Metabolic process	6	*Pkm, Me2, Cndp2, Pm20d1, Dlat, Cps1*
		Proton acceptor	3	
		Metal ion binding	5	
3	1.50	Extracellular matrix	4	*Pkm, P4hb, Cndp2, Pm20d1, Eef2, Cct6a, Cps1*
		Extracellular exosome	6	
		Poly(A) RNA binding	4	
		Nucleotide-binding	4	

[a]Biological processes associated with putative targets of DOWNREGULATED miRNAs in Tg villus-crypt axis
[b]Biological processes associated with putative targets of UPREGULATED miRNAs in Tg villus-crypt axis

Supplementary Table S6. Enriched functional clusters effected in both genotypes

Annotation Cluster[a]	Enrichment Score	Term	Count	Genes
1	1.6	Extracellular exosome	4	*Actb, Atic, Eef2, Calr*
		Acetylation	4	
		Cytosol	3	
		Membrane	4	
**Annotation Cluster[b]**	**Enrichment Score**	**Term**	**Count**	**Genes**
1	1.72	Smooth endoplasmic reticulum	3	*Pdia6, Hspa5, Canx*
		Melanosome	3	
		Protein processing in endoplasmic reticulum	3	
		Endoplasmic reticulum	3	
2	1.21	Extracellular exosome	6	*Actb, Taldo1, Ezr, Pdia6, Hspa5, Canx*
		Focal adhesion	3	

[a]Biological processes associated with putative targets of DOWNREGULATED miRNAs expressed in villus-crypt axis of WT and Tg
[b]Biological processes associated with putative targets of UPREGULATED miRNAs expressed in villus-crypt axis of WT and Tg
